# Mesenchymal stem cell origin contributes to the antitumor effect of oncolytic virus carriers

**DOI:** 10.1016/j.omton.2024.200896

**Published:** 2024-10-18

**Authors:** Makoto Sukegawa, Yoshitaka Miyagawa, Seiji Kuroda, Yoshiyuki Yamazaki, Motoko Yamamoto, Kumi Adachi, Hirofumi Sato, Yuriko Sato, Nobuhiko Taniai, Hiroshi Yoshida, Akihiro Umezawa, Mashito Sakai, Takashi Okada

**Affiliations:** 1 Department of Biochemistry and Molecular Biology, Graduate School of Medicine, Nippon Medical School, Tokyo, Japan; 2 Department of Gastrointestinal Surgery, Graduate School of Medicine, Nippon Medical School Musashikosugi Hospital, Kawasaki, Japan; 3 Department of Surgery, Graduate School of Medicine, Nippon Medical School, Tokyo, Japan; 4 Center for Regenerative Medicine, National Center for Child Health and Development Research Institute, Tokyo, Japan; 5 Division of Molecular and Medical Genetics, The Institute of Medical Science, The University of Tokyo, Tokyo, Japan

**Keywords:** MT: Regular Issue, mesenchymal stem cells, oncolytic herpes simplex virus, three-dimensional culture, cancer spheroids, OV-loaded MSCs

## Abstract

Oncolytic virotherapy shows promise as a cancer treatment approach; however, its systemic application is hindered by antibody neutralization. This issue can be overcome by using mesenchymal stem cells (MSCs) as carrier cells for oncolytic viruses (OVs). However, it remains elusive whether MSC source influences the antitumor effect. Here, we demonstrate that their source affects the migration ability and oncolytic activity of OV-loaded MSCs. Among human MSCs derived from different tissues, bone marrow-derived MSCs (BMMSCs) showed a high migration ability toward cancer cells in two- and three-dimensional MSC-cancer cell co-culture models. Comprehensive gene expression and Gene Ontology-based functional analyses suggested that genes involved in cell migration and cytokine response influence the cancer-specific tropism of BMMSCs. Furthermore, MSC origin affected the susceptibility to OVs, including cytotoxicity resistance and OV release from MSCs. MSC-mediated OV delivery significantly increased the viral spread and antitumor activity compared with delivery by OVs alone, and OV-loaded BMMSCs demonstrated the most potent antitumor effect among OV-loaded MSCs. Our results offer promising insights into cancer gene therapy with carrier cells and can help with the selection of an appropriate MSC source for MSC-based OV therapy.

## Graphical abstract

**Figure:**
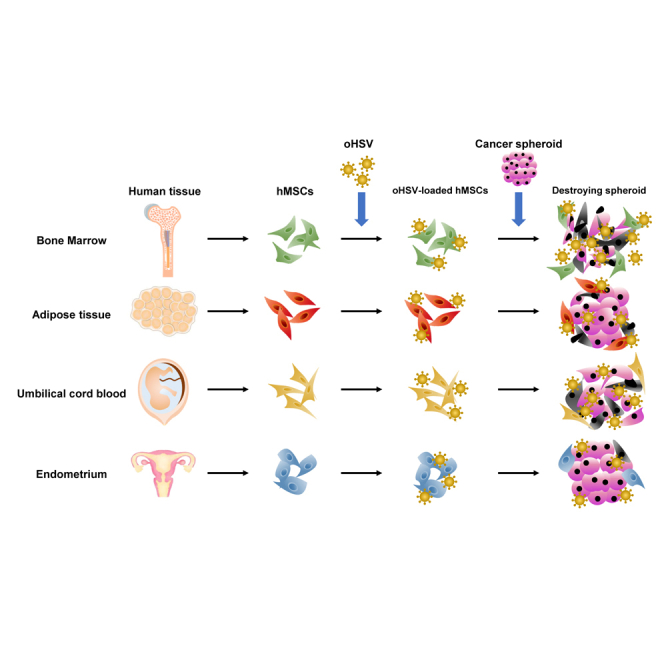

## Teaser

Miyagawa and colleagues unveil the varied performance of mesenchymal stem cells (MSCs) from different tissues as carriers for oncolytic viruses in two- and three-dimensional culture, suggesting bone marrow-derived MSCs as potentially the most effective for cancer treatment.

## Introduction

Oncolytic virotherapy has gained attention as a new approach to cancer treatment. Oncolytic viruses (OVs) can replicate and spread within tumor tissues, selectively killing tumors without harming normal cells. Advances in genetic recombination technology and the discovery of new molecular mechanisms of viral cytotoxicity have allowed for the enhancement of their antitumor effects. There are two known pathways through which OVs exert their antitumor effects^1^: they can directly destroy cancer cells^2^^,^^3^ or induce immunogenic cell death, activating dendritic cells along with tumor-associated antigens, ultimately eliciting effective antitumor immunity.^4^^,^^5^

Among the various OVs, several variants of the oncolytic herpes simplex virus (oHSV) have been extensively tested in clinical trials. These variants include NV1020,^6^ G207,^7^ talimogene laherparepvec (T-VEC, OncoVEX^GM−CSF^),^8^ HSV1716,^9^ and HF10.^10^ T-VEC is a genetically engineered oHSV that was approved by the Food and Drug Administration in 2015 for the treatment of melanoma. It harbors specific genetic modifications, including the deletions of ICP34.5 and ICP47, and the insertion of GM-CSF as a transgene. HF10, which was used in the present study, is a naturally occurring, highly attenuated, replication-competent mutant. UL43, UL49.5, UL55, UL56, and latency-associated transcripts are functionally deleted in HF10, which contains two copies of UL53 and UL54.^11^ HF10 showed high safety levels and oncolytic activity in pre-clinical studies^11^ and a clinical trial,^12^ suggesting that HF10 still has great potential for increase of its antitumor activity by genetic engineering. Therefore, we questioned whether the antitumor activity of HF10 can be improved by using carrier cells. The advantages of oHSV include its relative ease of genome manipulation, large coding capacity for transgene insertion, the ability to target specific cell receptors by altering surface glycoproteins, and the ability to control replication with herpes virus-specific drugs such as acyclovir.^10^ Therefore, oHSV is considered a promising option among available OVs.

Nonetheless, based on clinical experience, OV monotherapy has shown limited antitumor effects to date.^13^ First, OVs may fail to replicate in and lyse tumor cells because of the presence of pre-existing or treatment-induced neutralizing antiviral antibodies.^14^ Second, the tumor microenvironment (TME), which consists of cancer-associated fibroblasts (CAFs), immune cells, and endothelial cells, can hinder OVs from accessing cellular entry receptors expressed in tight junctions.^15^ To enhance the antitumor effects, various combinations of OVs with other antitumor agents, including radiotherapy, chemotherapy, immune checkpoint inhibitors, and chimeric antigen receptor T cell therapies, have been evaluated.^16^ In addition, genetic modification of OVs has also been explored.^1^ However, most of these approaches failed to substantially improve the therapeutic effect.^17^^,^^18^

The use of carrier cells for OVs has gained attention as a solution for the insufficient antitumor activity of OVs.^19^ Carrier cells can protect OVs from the immune system, leading to improved antitumor efficacy. Various cells, including mesenchymal stem cells (MSCs), neural stem cells, monocytes, and T lymphocytes, have been reported as promising carrier cells because of their attributes. Particularly, MSCs display excellent properties as carrier cells due to their superior functions, including immunotolerance,^20^ tumor-homing ability,^21^ and tumor invasiveness.^22^

Some clinical trials (NCT02068794, NCT01844661) have investigated MSCs as carrier cells for OVs, with promising results.^23^^,^^24^ Although the application of MSCs as viral carriers has progressed, some issues regarding effectiveness and administration remain elusive.^25^ One remaining challenge is the selection of an appropriate MSC source. MSCs can be isolated from a wide range of human tissues, such as bone marrow, umbilical cord blood, and adipose. However, MSCs differ in terms of cell morphology, proliferation, immunophenotype, immunomodulatory function, and differentiation capacity depending on their source.^26^ Consequently, their susceptibility to viruses and accumulation in tumor tissues can vary. Therefore, it is necessary to select the most suitable MSC source for the development of OV carriers and therapeutic targets.

To identify an appropriate MSC source for MSC-based OV therapy, we compared the characteristics of MSCs derived from various human tissues as OV carriers in a two-dimensional (2D) culture system. Furthermore, we established a three-dimensional (3D) co-culture model that mimicked the tumor environment *in vivo* and evaluated the characteristics of MSCs as OV carriers using this model. We demonstrated that the origin of MSCs influences their functionality as carrier cells, including their homing ability to cancer cells, reactivity to OVs, and tumor-killing ability when loaded with OVs. Our findings emphasize the importance of selecting the appropriate source of MSCs.

## Results

### BMMSCs have superior migration ability toward cancer cells compared with other human MSCs in 2D culture

**Figure 1:**
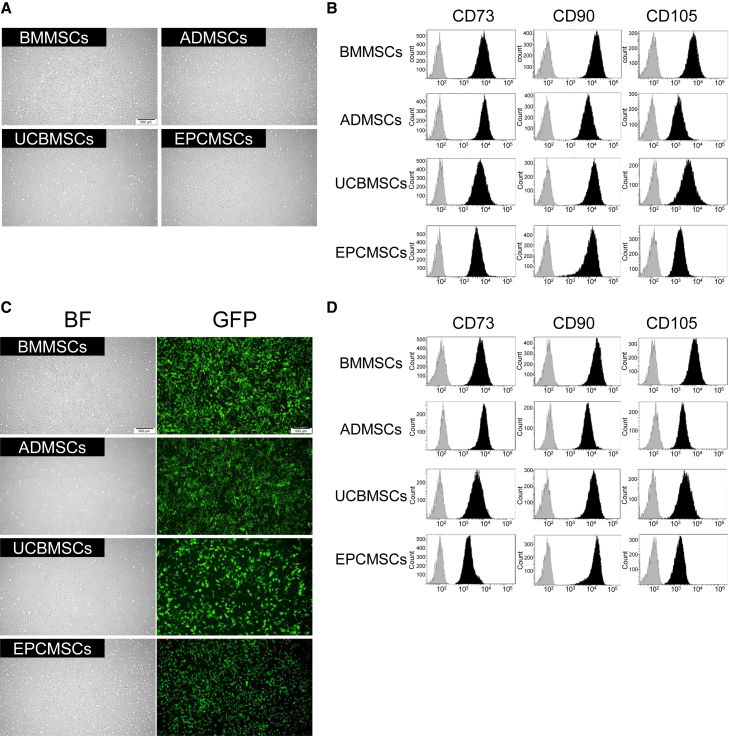
Morphology and surface antigen expression of the hMSCs used in this study (A) Phase-contrast photomicrographs of BMMSCs, ADMSCs, UCBMSCs, and EPCMSCs. Scale bar, 500 μm. (B) Flow cytometry analysis of CD73, CD90, and CD105 expression on hMSCs. Light gray graphs represent cells stained with isotype control antibodies, and black graphs represent cells stained with individual antibodies. (C) Photomicrographs of hMSCs expressing AcGFP through lentiviral infection. Phase-contrast images (bright field: BF, left) and AcGFP fluorescence images (GFP, right) are shown. Scale bars, 500 μm. (D) Flow cytometry analysis of hMSC marker expression on hMSCs expressing AcGFP.

**Figure 2:**
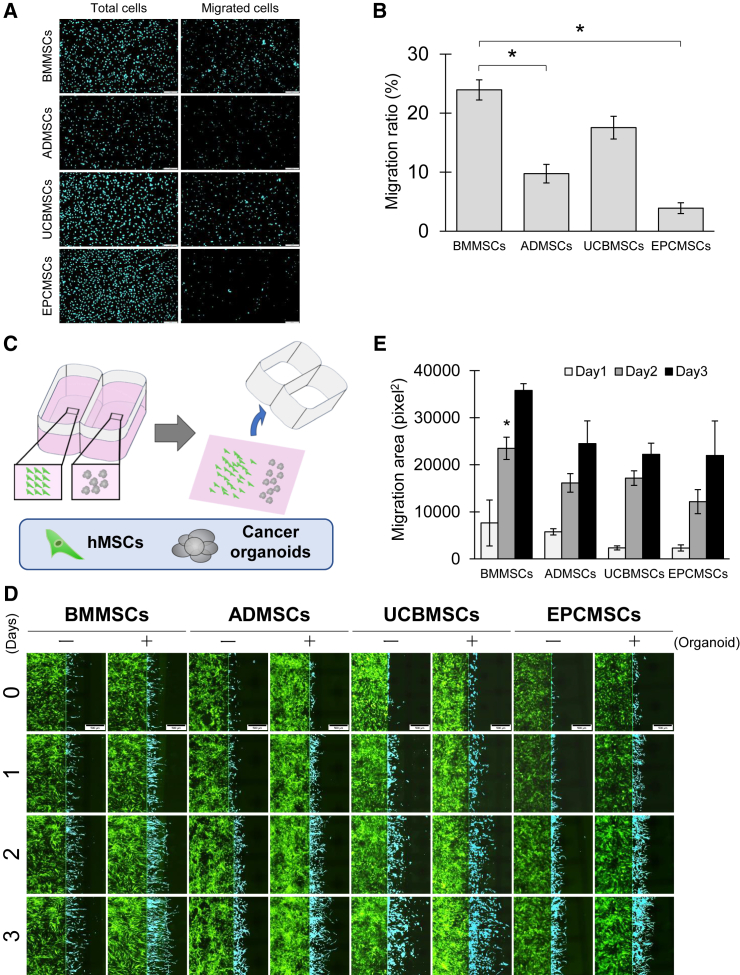
Evaluation of cancer-specific tropism of hMSCs in 2D culture models (A) hMSC migration assay toward PANC1-CM. hMSCs were seeded in the upper chamber of the Transwell, whereas PANC1-CM was added in the lower chamber. Scale bars, 200 μm. Next, 24 h after hMSC seeding, hMSC nuclei were stained with Hoechst 33342 and the fluorescence was photographed. (B) Migration ratio of hMSCs toward PANC1-CM in the vertical migration assay. Cells were counted in five random microscope fields for each sample in three independent experiments. Cell numbers relate to arbitrary relative units of Transwell-migrating hMSCs. Data represent the mean ± SEM. Differences between BMMSCs and ADMSCs and between BMMSCs and EPCMSCs were statistically significant (∗*p* < 0.05). (C) Schema of the assay of hMSC horizontal migration toward bile duct cancer organoids. To divide hMSCs and cancer organoids, hMSCs were seeded on the left side of the two-well culture insert (80209; Ibidi), whereas cancer organoids were placed on the right side. Next, 24 h after seeding, the insert was removed, and the hMSCs were allowed to migrate toward the cancer organoids. (D) Assay of hMSC migration toward bile duct cancer organoids. Fluorescence images were acquired daily. Left columns (−) represent micrographs of hMSCs cultured without cancer organoids as control groups, whereas right columns (+) show micrographs of hMSCs co-cultured with cancer organoids. hMSCs are labeled green, and migrated cells are shown in light-blue color. (E) The migration efficiency of hMSCs toward cancer organoids. The area of pixels occupied by migrated hMSCs was measured using the Image-PRO image analysis software (Hakuto), and differences in migrated hMSC areas between organoid (+) and (−) groups were calculated. Data represent the mean ± SEM. The difference between BMMSCs and EPCMSCs on day 2 was statistically significant (∗*p* < 0.05, one-way ANOVA followed by Tukey’s multiple comparisons test). The assay was conducted in triplicate. Scale bars, 500 μm.

To compare the characteristics of human MSCs (hMSCs), we prepared immortalized hMSCs sourced from the bone marrow (BMMSCs), adipose (ADMSCs), umbilical cord blood (UCBMSCs), and endometrium (EPCMSCs) (Figure 1A), expressing the representative mesenchymal markers CD73, CD90, and CD105 (Figure 1B). To monitor hMSC mobility in 2D culture systems, the hMSCs were labeled with *Aequorea coerulescens* green fluorescent protein (AcGFP) through lentiviral transduction (Figure 1C). The AcGFP transduction did not affect the mesenchymal marker expression levels (Figure 1D). To assess the migration ability of hMSCs toward cancer cells, we initially conducted vertical migration assays using Transwells (Figure 2). As expected, all four types of hMSCs migrated toward pancreatic cancer cell-conditioned medium (PANC-CM) in Transwells (Figure 2A). Meanwhile, only a small number of cells migrated toward human dermal fibroblast cell-conditioned medium (HDF-CM) (Figure S1A). Interestingly, the number of cells that migrated toward PANC-CM varied among the different types of hMSCs. BMMSCs showed superior migration ability, whereas EPCMSCs exhibited inferior migration ability (Figure 2B). Next, horizontal migration assays were conducted to monitor MSC migration toward human bile duct cancer organoids over time (Figures 2C and S2). Consistent with the vertical migration assay results, all hMSCs migrated spontaneously even in the absence of cancer organoids (Figure S1B), but their migration toward cancer organoids was remarkable (Figure 2D), suggesting that hMSCs can spontaneously home cancer organoids. BMMSCs had the highest efficiency (Figure 2E). Together, these results indicated that their source influences the migratory potential of hMSCs toward cancer cells in a 2D culture system.

### BMMSCs have superior migration ability toward cancer cells compared with other hMSCs in 3D culture

**Figure 3:**
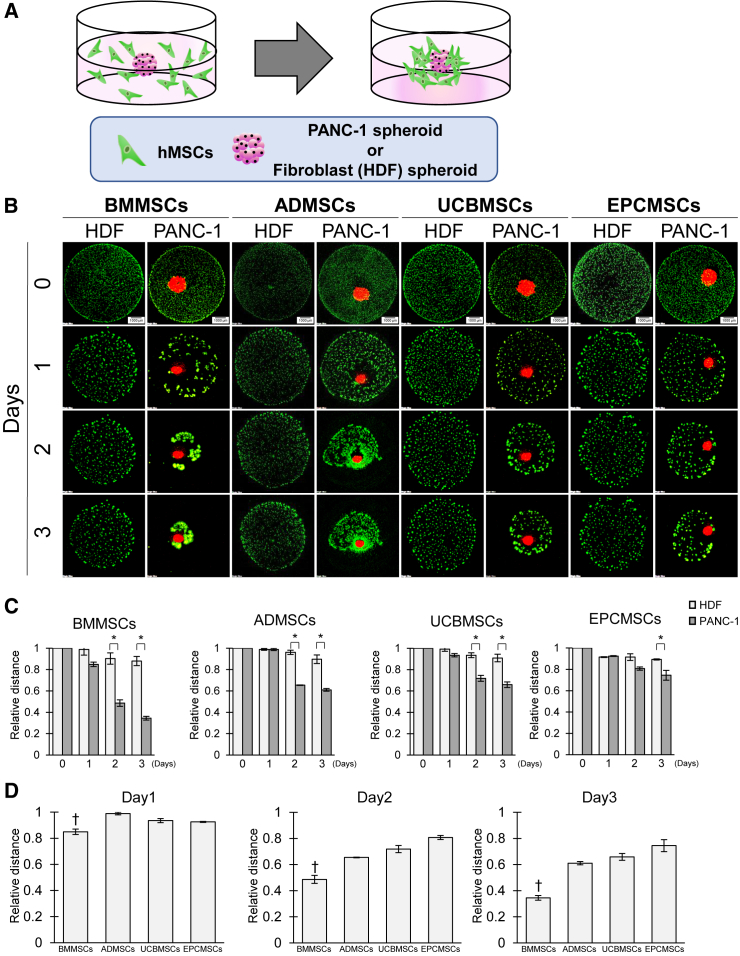
Evaluation of cancer-specific tropism of hMSCs in a 3D co-culture model (A) Schema of the assay of hMSC 3D migration toward a PANC-1 spheroid. hMSCs were suspended in serum-free medium containing Matrigel and seeded on a low-attachment plate. Then, a PANC-1 or HDF spheroid was placed at the center of the well. (B) Monitoring of hMSC migration toward a PANC-1 spheroid in 3D co-culture. Confocal z stack images (step size 3 μm, 55× slices, 40× objective) were acquired daily using a confocal microscope (FV1200, Olympus). The micrographs are shown as maximum-intensity projections. The left columns show micrographs of hMSCs with an HDF spheroid as control groups, and the right columns show micrographs of hMSCs with a PANC-1 spheroid. hMSCs, green; PANC-1, red. HDF spheroids were not labeled. Bright-field images of HDF groups are provided in Figure S3. Scale bars, 1,000 μm. (C) Migration ratio of hMSCs toward a spheroid in 3D co-culture. The distance between hMSCs and a PANC-1 or HDF spheroid was measured daily using the Image-PRO software. The migration ratio was calculated relative to the distance on day 0. Light-gray bars show the migration ratio of hMSCs toward an HDF spheroid, and dark gray bars show the migration ratio of hMSCs toward a PANC-1 spheroid. All four types of hMSCs showed notable migration ability toward a spheroid formed with PANC-1 cells compared with HDF (∗*p* < 0.05, one-way ANOVA followed by Tukey’s multiple comparisons test). (D) The relative distance between hMSCs and a PANC-1 spheroid on each day. Differences between BMMSCs and other hMSCs were statistically significant (†*p* < 0.05, one-way ANOVA followed by Tukey’s multiple comparisons test). Data represent the mean ± SEM. The assay was conducted in triplicate.

Studies have demonstrated that 2D culture systems do not fully recapitulate the TME,^27^^,^^28^^,^^29^^,^^30^^,^^31^ limiting the proper monitoring of migration abilities and therapeutic effects.^32^^,^^33^ Therefore, we established a 3D co-culture model to assess hMSC characteristics in the TME. To monitor the migration ability of hMSCs toward cancer cells, we placed an mCherry-expressing cancer spheroid derived from a human pancreatic cancer cell line PANC-1 at the center of each well of a low-attachment plate and then plated hMSCs in hMSC basal medium containing 5% Matrigel (Figures 3A and S3). Human dermal fibroblast-derived spheroids were used as a negative control (denoted as “HDF”). In this culture system, the 3D dynamics of hMSC can be quantitatively monitored over time (Figure 3B). The PANC-1 spheroid seemed to spontaneously shrink in size over time in this 3D migration assay (Figure S4), as described previously for other cancer spheroids.^34^^,^^35^ hMSCs remarkably migrated toward PANC-1 spheroids, whereas their migration toward HDF spheroids was minimal, suggesting that hMSC migration is cancer-specific even in 3D culture settings. The migration ability is independent of the source tissue (Figure 3C). Of note, the accumulation pattern of the hMSCs around the cancer spheroids varied greatly depending on their source. For instance, BMMSCs rapidly formed small aggregates before migrating toward the spheroids and accumulating around the cancer spheroids. In contrast, ADMSCs migrated without forming aggregates (Figure 3B). BMMSCs migrated more rapidly toward the spheroids than the other hMSCs (*p* < 0.05; Figure 3D). Similarly, primary BMMSCs demonstrated tumor-specific migration in the 3D co-culture system (Figure S5). We also performed 3D migration assays with spheroids derived from other cancer cell lines (Figure S6). BMMSCs migrated more rapidly toward DLD-1 and T24 spheroids than toward HDF spheroids, whereas the migration toward U2OS spheroids was not significant, indicating that the extent of migration seemed to depend on the type of cancer cells. These results indicated that BMMSCs exhibit stronger cancer tropism than other hMSCs, not only in the 2D system but also in the 3D system. To evaluate whether the migration ability of BMMSCs toward tumors is superior to others *in vivo*, we tested the migration of BMMSCs and EPCMSCs toward the tumor in a pancreatic cancer xenograft model (Figure S7). Consistent with the results in our 3D co-culture model, the accumulation of BMMSCs at tumor sites was significantly higher than that of EPCMSCs, indicating that our 3D co-culture model is useful to predict the migration ability of hMSCs toward tumors *in vivo*.

### Comprehensive gene expression analysis of hMSCs reveals key regulators of migration toward cancer cells

**Figure 4:**
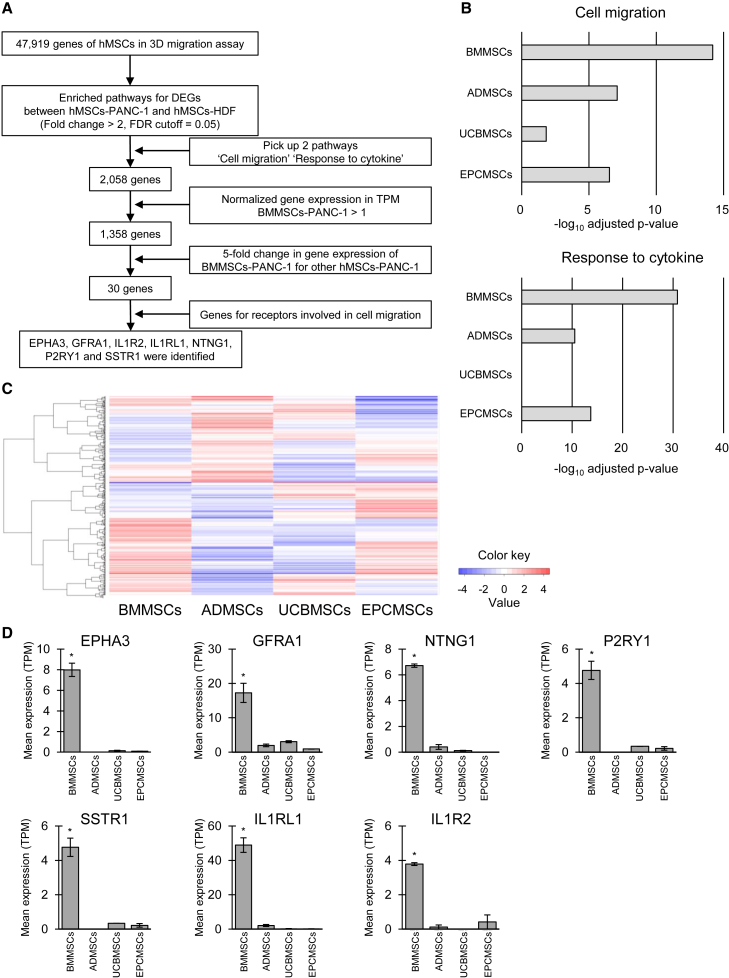
Comprehensive gene expression analysis of hMSCs in a 3D co-culture model (A) Strategy used to identify expressed genes related to migration. We started with an initial pool of 47,919 genes. By focusing on two enriched pathways, 2,058 genes were identified. Of these, 1,358 genes were strongly expressed in BMMSCs. Notably, 30 genes showed at least a 5-fold increase in expression between BMMSCs-PANC and other hMSCs-PANC. Finally, seven candidate genes involved in cancer-specific tropism in BMMSCs were selected. (B) GO terms enriched in hMSCs. The enrichment scores [–log_10_(*p* value)] for the four types of hMSCs for the GO terms “cell migration” (GO: 0016477) and “response to cytokine” (GO: 0034097) are shown. (C) Heatmap of gene expression patterns in the hMSCs. Red indicates upregulation, and blue indicates downregulation. (D) Expression levels of candidate genes involved in the cancer-specific tropism in BMMSCs. Expression levels were normalized relative to tpm. ∗*p-adj* < 0.05. The assay was conducted in duplicate.

The results above suggest that the origin of hMSCs did affect cancer tropism in our co-culture models. To investigate the underlying mechanism, we conducted a comprehensive RNA-sequencing-based gene expression analysis of the 3D co-culture models using four types of hMSCs (Figure 4A). In total, we detected 47,919 genes in the hMSCs via RNA sequencing. Differentially expressed genes (DEGs) were identified based on a fold change >2 and a false discovery rate cutoff of 0.05 and subjected to Gene Ontology (GO) functional analysis. DEGs between hMSC-PANC1 and hMSC-HDF spheroid co-cultures were enriched in two biological pathways: “cell migration” (GO: 0016477) and “response to cytokine” (GO: 0034097). These pathways encompassed 2,058 genes. Among these, 1,358 genes had normalized expression values >1 in BMMSC-PANC1 (transcripts per million > 1). From these genes, we selected those that showed a more than a 5-fold increase in expression in BMMSC-PANC1 spheroid co-culture compared with that in other hMSC-PANC1 spheroid co-cultures, yielding 30 genes. Among these, *EPHA3*, *GFRA1*, *IL1R2*, *IL1RL1*, *NTNG1*, *P2RY1*, and *SSTR1* regulate receptor expression associated with cell motility. GO analysis revealed that both pathways showed small *p* values in BMMSCs-PANC1 spheroid co-culture (Figure 4B), suggesting the upregulation of these genes and, consequently, an enhancement of cell migration and the response to cytokines. The gene expression heatmap in Figure 4C visualizes the differences in gene expression patterns among the four types of hMSCs. Notably, the seven genes associated with cell motility exhibited particularly higher expression in BMMSCs than that in the other hMSCs (Figure 4D). These results suggested that BMMSCs may regulate the expression of specific genes to enhance their ability to migrate toward cancer cells. Specifically, these seven genes have the potential to play pivotal roles in this process. Among those seven genes, we focused on SSTR1 because somatostatin (SST) has been reported to be associated with cell migration.^36^^,^^37^^,^^38^^,^^39^ The gene expression level of hMSCs in 2D culture was evaluated using real-time quantitative polymerase chain reaction (qRT-PCR) (Figure S8). BMMSCs showed higher expression levels of SSTR1 compared with other hMSCs. Notably, the migration of BMMSCs was inhibited by the SSTR1 antagonist, CYN154806 (Figure S9). These results suggest that SSTR1 can, at least in part, play an important role in hMSC migration toward cancer spheroids.

### hMSC source influences the susceptibility to oHSVs

**Figure 5:**
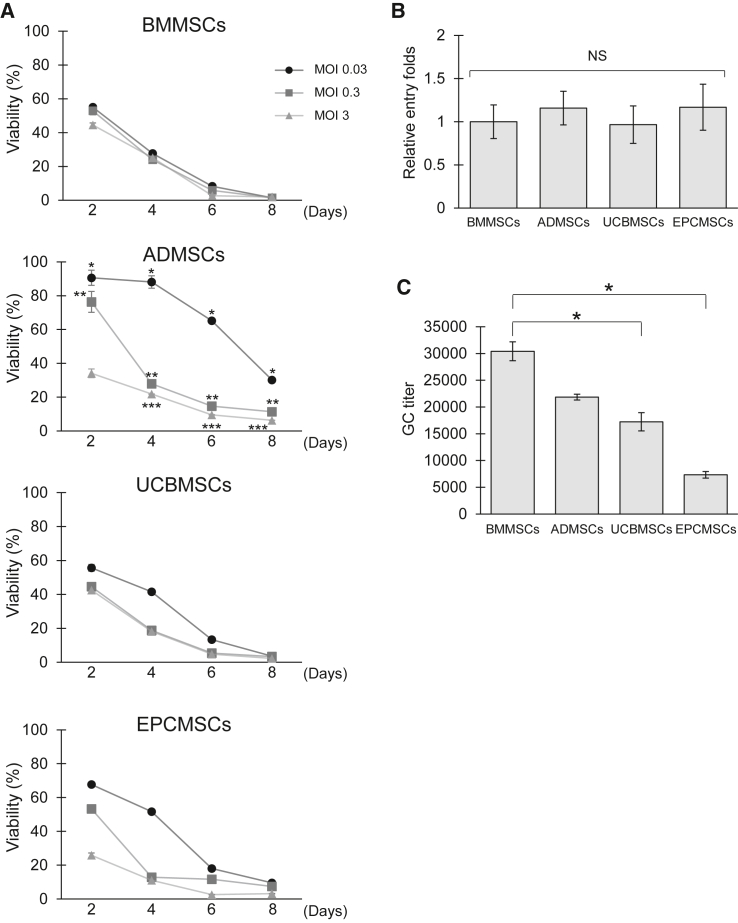
Behavior of oHSV in hMSCs, including toxicity, viral entry, and release efficiency (A) Viability of hMSCs infected with oHSVs at various MOIs. hMSCs were infected with oHSVs at an MOI of 0.03 (●), 0.3 (▪), and 3 (▲). The viability of infected hMSCs was determined by MTT assay at the indicated time points and calculated as a percentage relative to non-infected control cells. Data represent the mean ± SEM. Differences between ADMSCs and other hMSCs at all of the MOIs were statistically significant (∗*p* < 0.01 for MOI 0.03, ∗∗*p* < 0.01 for MOI 0.3, ∗∗∗*p* < 0.05 for MOI 3, one-way ANOVA followed by Tukey’s multiple comparisons test). (B) The viral entry efficiency of oHSV to hMSCs. hMSCs were infected with oHSVs at an MOI of 2 for 2 h, the cells were harvested, and the nuclei were purified. The physical titer of oHSV in hMSC nuclei was determined using qPCR as described in materials and methods. The viral entry efficiency was calculated as the ratio of oHSV GC in hMSC nuclei to that in BMMSCs. Data represent the mean ± SEM. (C) The viral release efficiency of oHSV from hMSCs. hMSCs were infected at an MOI of 2 for 2 h, the supernatants were replaced with fresh medium, and the cells were cultured for 2 days. The supernatants were collected, and the oHSV GC was determined using qPCR. Data represent the mean ± SEM. Differences between BMMSCs and UCBMSCs and between BMMSCs and EPCMSCs were statistically significant (∗*p* < 0.05, one-way ANOVA followed by Tukey’s multiple comparisons test). The assay was conducted in triplicate.

When utilizing hMSCs as carrier cells for oHSVs, the susceptibility to oHSVs is crucial. Hence, we first focused on the resistance to oHSV (Figure 5A). All hMSCs were highly sensitive to oHSV *in vitro*, leading to rapid cell death within 48 h, but BMMSCs were less resistant to oHSV. Interestingly, ADMSCs were somewhat resistant to oHSV infection, suggesting that their origin affects the susceptibility of hMSCs to oHSV. To elucidate the cause of the susceptibility, we compared the infection efficiency of oHSV among the hMSCs (Figure 5B), but we found no notable differences. We also evaluated the efficiency of oHSV release from hMSCs after oHSV infection (Figure 5C). oHSV-loaded BMMSCs (oHSV-BMMSCs) significantly secreted oHSVs in the culture supernatant compared with oHSV-loaded UCBMSCs (oHSV-UCBMSCs) (*p* < 0.05) and oHSV-loaded EPCMSCs (oHSV-EPCMSCs) (*p* < 0.05), suggesting that high-level secretion of oHSVs can contribute to BMMSC susceptibility to oHSV. However, no considerable difference was observed in oHSV release efficiency between BMMSCs and ADMSCs. This finding demonstrated that the low susceptibility of ADMSCs to oHSV does not correlate with the infection and replication efficiency and may be influenced by tissue-specific mechanisms.

### oHSV-loaded hMSCs efficiently disseminate into PANC-1 cells in 2D culture

**Figure 6:**
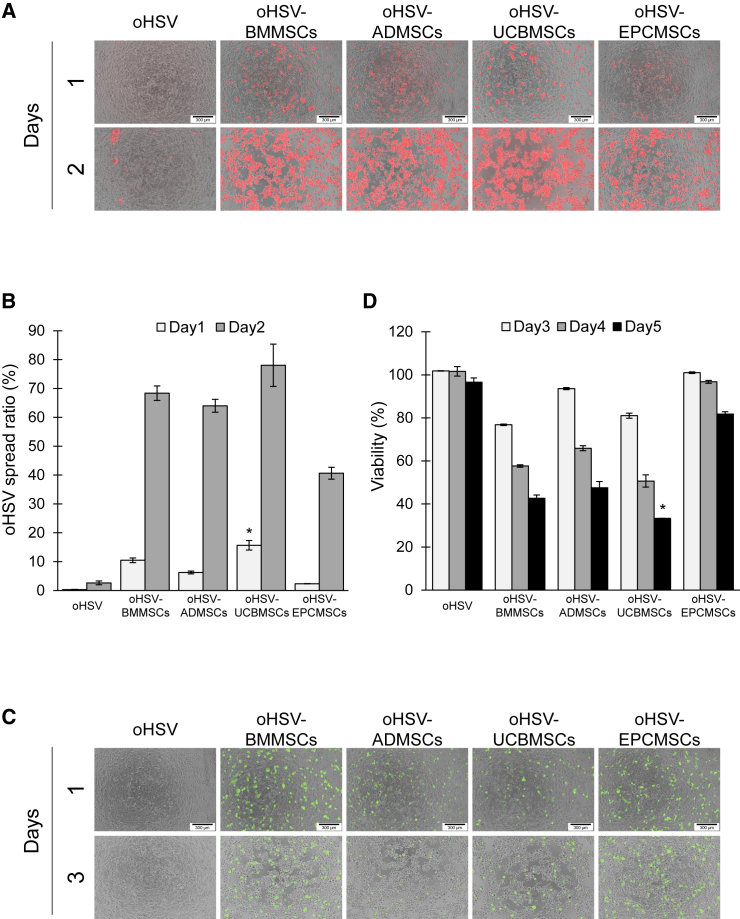
Spread and killing activity of oHSVs and oHSV-hMSCs in PANC-1 cells in a 2D co-culture model (A) Spread of oHSVs into PANC-1 cells in a 2D co-culture model. PANC-1 cells were infected with oHSV expressing mCherry or co-cultured with oHSV-hMSCs, and the fluorescence was photographed daily. PANC-1 cells; non-labeled, oHSV; red. Scale bars, 300 μm. (B) The spread ratio of oHSV into PANC-1 cells in a 2D co-culture model. oHSV spread ratio was calculated as a ratio of mCherry-positive cells to all cells. Data represent the mean ± SEM. Differences between UCBMSCs and other hMSCs were statistically significant (∗*p* < 0.05, one-way ANOVA followed by Tukey’s multiple comparisons test). (C) The micrographs of the PANC-1 cells infected with oHSV or co-cultured with oHSV-hMSCs in a 2D co-culture model. The cell morphology and fluorescence were photographed daily. PANC-1 cells; non-labeled, hMSCs; green. Scale bars, 300 μm. (D) The viability of PANC-1 cells infected with oHSV in a 2D co-culture model. The viability of PANC-1 cells was determined by CCK-8 assay on days 3, 4, and 5 after infection. The viability ratio was calculated as a percentage of non-infected control cells. Data represent the mean ± SEM. Differences between UCBMSCs and other hMSCs on day 5 were statistically significant (∗*p* < 0.05, one-way ANOVA followed by Tukey’s multiple comparisons test). The assay was conducted in triplicate.

An important consideration for oHSV delivery vehicles is their ability to efficiently deliver oHSV into cancer cells.^40^ Therefore, we next examined whether using hMSCs as oHSV carriers could enhance the viral spread and killing activity toward cancer cells using the 2D co-culture model (Figure 6). mCherry-expressing oHSV or oHSV-loaded hMSCs (oHSV-hMSCs) were co-cultured with a monolayer of PANC-1 cells, and the viral spread in the cells and the cytotoxicity of oHSV-hMSCs toward PANC-1 cells were measured. oHSV delivered by hMSCs showed a higher diffusion capability than oHSV alone (*p* < 0.05) (Figures 6A and 6B). In the oHSV-alone group, few PANC-1 cells were mCherry positive on day 2, whereas in the oHSV-hMSCs groups most PANC-1 cells had a round shape and expressed mCherry, suggesting that oHSV had spread throughout the cells (Figures 6A and S10). We next evaluated the cytotoxicity of oHSV-hMSCs toward PANC-1 cells using the 2D co-culture model (Figures 6C and 6D). On day 5, >90% of PANC-1 cells were alive after oHSV infection alone, whereas >50% of PANC-1 cells were killed by oHSV-hMSCs, except for oHSV-EPCMSCs. PANC-1 cells treated with oHSV-UCBMSCs showed the lowest viability on days 3 (81%), 4 (50.7%), and 5 (33.3%), whereas oHSV-EPCMSCs showed the lowest killing ability. We also determined the viability of PANC-1 cells on day 2 by flow cytometry, staining dead cells with Zombie NIR viability dye. Similar results were obtained in terms of mortality trends (Figure S11). These data suggested that hMSCs facilitated efficient oHSV spread and antitumor activity in the 2D co-culture model.

### oHSV-hMSCs, particularly oHSV-BMMSCs, enhance the viral spread and the antitumor effect in PANC-1 spheroids in 3D culture

**Figure 7:**
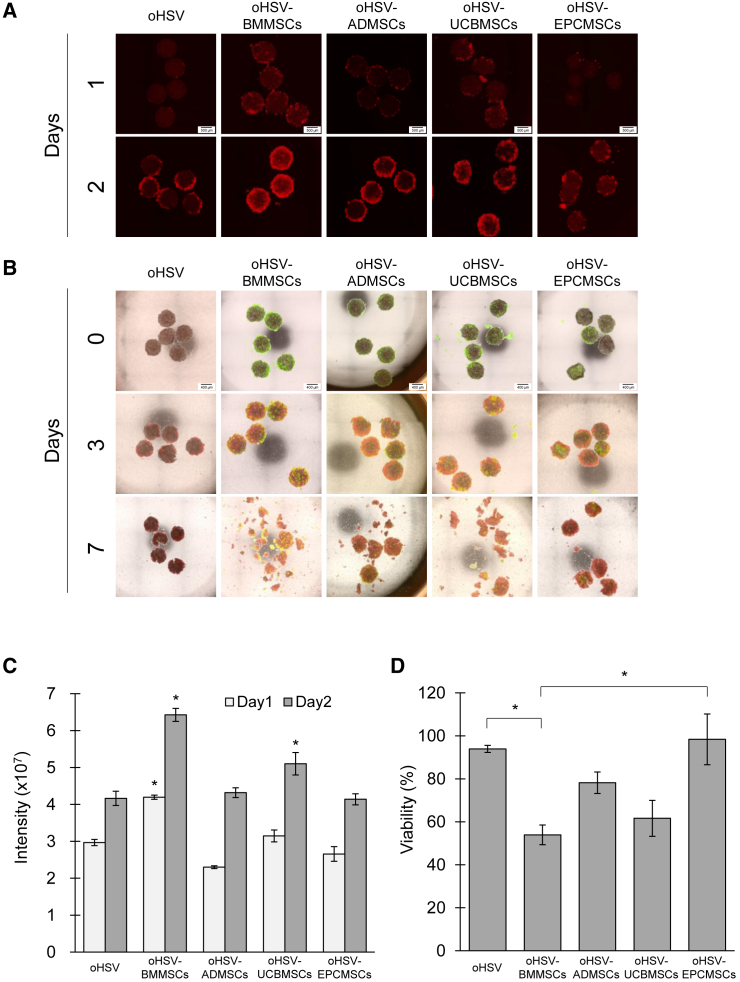
Spread and killing activity of oHSVs in PANC-1 spheroids in a 3D co-culture model (A) Spread of oHSVs in PANC-1 spheroids in a 3D co-culture model. Five PANC-1 spheroids and oHSVs or oHSV-hMSCs were cultured under horizontal rotation at 37°C for 2 h, resuspended in 5% Matrigel, and seeded in a low-attachment plate. Micrographs were captured daily. oHSV-infected cells: red. Scale bars, 500 μm. (B) Micrographs of PANC-1 spheroids infected with oHSVs or co-cultured with oHSV-hMSCs in a 3D co-culture model. hMSCs, green; oHSV-infected cells, red. Scale bars, 400 μm. (C) Spread efficiency of oHSVs in PANC-1 spheroids. The fluorescence intensity in PANC-1 spheroids infected with oHSVs was measured using the Image-PRO software. Data represent the mean ± SEM. Differences between BMMSCs and other hMSCs were statistically significant (∗*p* < 0.05, one-way ANOVA followed by Tukey’s multiple comparisons test). (D) Viability of PANC-1 spheroids in a 3D co-culture model. The viability of PANC-1 spheroids was determined using a CCK-8 assay and calculated as a percentage of that of non-infected PANC-1 spheroids. Data represent the mean ± SEM. Differences between BMMSCs and oHSVs and between BMMSCs and EPCMSCs were statistically significant (∗*p* < 0.05, one-way ANOVA followed by Tukey’s multiple comparisons test). The assay was conducted in triplicate.

Finally, we examined the viral spread efficacy and the killing ability of oHSV loaded on hMSCs in cancer cells using the 3D co-culture model mimicking the tumor environment *in vivo*. To evaluate the migration ability of oHSV-hMSCs, the 3D migration assay for oHSV-BMMSCs was performed. However, they did not show migration toward a cancer spheroid (Figure S12), because, as shown in Figure 5, hMSCs are killed by oHSV after infection over time, whereas it takes about 3 days for hMSCs to reach tumor spheroids after seeding (Figure 3). To check oHSV-hMSC dynamics after migration to the tumor, oHSV-BMMSCs were attached to the spheroids in advance. To allow efficient attachment of the oHSV-hMSCs to the PANC-1 spheroids, we first evaluated the rotation co-culture conditions, including the numbers of spheroids and hMSCs, cultivation methods, and temperature, using oHSV-hMSCs and PANC-1 spheroids (Figure S13), referring to previous publications.^41^^,^^42^^,^^43^ We found an optimal rotation culture of five PANC-1 spheroids and 2 × 10^5^ hMSCs in 125 μL of medium at 36 rpm. Under these conditions, oHSV-hMSCs are efficiently attached to PANC-1 spheroids. After the oHSV-hMSCs had been attached to the PANC-1 spheroids, they were seeded into 3D cultures to investigate the spread of oHSVs in the PANC-1 spheroids. mCherry signals spread in the cancer spheroids over time (Figure 7A). By observing the middle slice of the spheroid, we could confirm the infiltration of oHSV and hMSCs into the inside of the spheroid. The fluorescence merged images showed that GFP-positive hMSCs slightly infiltrated into the PANC-1 spheroid 6 h after infection (Figure S14, upper panel). Meanwhile, many GFP-negative/mCherry-positive cells were observed in the middle of the spheroid 72 h after infection (Figure S14, lower panel), indicating that oHSV had spread into the tumor spheroid (Figure S14). Although mCherry diffusion was observed for both oHSV-hMSCs and oHSV alone, the spread was markedly enhanced when BMMSCs and UCBMSCs were used as carrier cells (Figures 7A and 7B). Among the four types of oHSV-hMSCs, oHSV-BMMSCs demonstrated a superior ability to increase the oHSV spread efficiency in PANC-1 spheroids (*p* < 0.05; Figure 7C). We next explored cancer cell mortality in the 3D co-culture model (Figures 7B and 7D). On day 3, there were no noticeable changes in the morphology of PANC-1 spheroids in both the oHSV and oHSV-hMSCs groups. However, on day 7, the spheroids started to collapse, particularly in the oHSV-hMSCs groups (Figure 7B). More than 90% of PANC-1 spheroids remained alive in the oHSV and oHSV-EPCMSCs groups, whereas less than 60% remained alive in the oHSV-BMMSCs group. The results suggested that using BMMSCs as carrier cells enhanced the antitumor activity (Figure 7D). Overall, these findings indicated that hMSCs, with the exception of EPCMSCs, can be beneficial in promoting the spread and antitumor activity of oHSV in a 3D co-culture model.

## Discussion

Studies have been conducted to determine if OV-loaded MSCs exert more effective antitumor effects compared with OV monotherapy.^44^^,^^45^^,^^46^ It has been reported that MSCs effectively deliver different types of OVs, including oncolytic adenovirus, HSV, measles virus, myxoma virus, and reovirus, to the target site and enhance the antitumor efficacy.^47^ However, few studies have investigated whether their origin influences the functionality of MSCs as OV carrier cells. This study revealed the influence of the MSC source on their capability to function as oHSV carriers, including tumor homing, sensitivity to oHSV, and tumor-killing activity. We anticipate that hMSC origin is a critical factor in the selection of appropriate hMSCs as oHSV carrier cells.

Although the migration ability of MSCs to tumor cells and chemoattractants has been investigated, experiments were generally performed using conventional Transwell or Boyden chambers in 2D culture. The major limitation of vertical migration assay systems is the lack of cell-cell and cell-matrix interactions.^48^ In addition, monitoring mortality is challenging in these systems.^49^ In contrast, horizontal migration assay systems using Ibidi inserts allow cell-cell interactions and monitoring of cell migration over time. Furthermore, cells can form well-defined monolayer margins and edges without physical damage and migrate as a collective or detach from the edges and migrate as individual cells.^50^ We successfully observed the sequential motions of hMSCs in 2D culture, revealing the tumor-specific tropism of hMSCs and that BMMSCs can rapidly migrate toward cancer organoids even in the presence of cell-cell interaction (Figures 2C–2E). To further evaluate the hMSC migration ability in a 3D environment recapitulating tumor tissue, we developed a 3D co-culture model that allows cell-matrix interaction and enables the monitoring of MSC 3D dynamics during their migration to a tumor spheroid in a time-dependent manner (Figure 3). The 3D monitoring revealed the unique motility of each type of MSC, which could not be observed using the 2D co-culture model, and the high migration capacity of BMMSCs to tumor spheroids, suggesting that hMSC source influences cancer tropism in a 3D co-culture model. Consistent with the results in the 3D co-culture model, BMMSCs showed superior migration ability toward a cancer spheroid compared with EPCMSCs on a pancreatic cancer xenograft model (Figure S7), suggesting that our 3D co-culture model is useful for predicting the ability of hMSCs to migrate toward tumors *in vivo*. Our data indicate that 3D co-culture systems are an attractive alternative to 2D systems for monitoring MSC motility and tropism in tumor cells. Further improvement of the 3D co-culture system, including the addition of immune cells and CAFs, would be helpful to evaluate the properties of MSCs as OV carrier cells in more detail.

The expression and functionality of adhesion molecules, chemokine receptors, and metalloproteinase enzymes are vital in enabling MSC trafficking from the peripheral blood to specific target organs.^51^ MSCs demonstrate variable expression of chemokine receptors such as CCR2, 3, 4, 7, and 10, and CXCR4, 5, and 6, which participate in their movement and localization.^52^^,^^53^ In particular, CXCR4 is essential for regulating the homing and migration of hematopoietic stem cells and likely that of MSCs.^53^ Compared with hematopoietic stem cells, MSCs sporadically express CXCR4 on their surface. However, CXCR4 expression on MSCs can be enhanced via stimulation with inflammatory cytokines such as tumor necrosis factor α and interleukin-1β (IL-1β).^54^ Importantly, MSCs genetically engineered for enhanced CXCR4 production have demonstrated improved homing and migration capabilities in preclinical studies.^55^ We performed a comprehensive gene expression analysis, assuming that differences in gene expression were responsible for the superior migration ability of BMMSCs. Of note, we did not find significant differences in the expression of CCR2, 3, 4, 7, and 10, and CXCR4, 5, and 6 among the four types of hMSCs. Therefore, we attempted to identify factors related to MSC migration, and we found that DEGs associated with “cell migration” (GO: 0016477) and “response to cytokine” (GO: 0034097) were enriched. The expression of chemokine receptor genes involved in migration, i.e., *EPHA3*, *GFRA1*, *NTNG1*, *P2RY1*, *SSTR1*, *IL1R2*, and *IL1RL1*, was significantly upregulated in BMMSCs. For example, EPHA3 interacts with the receptors for ephrin, promoting the migration of colorectal epithelial cells,^56^ whereas IL1RL1 is involved with the IL-33 receptor attracting gastric cancer cells.^57^ SSTR1 is one of the somatostatin (SST) receptors, and SST has been reported to be associated with cell migration.^36^^,^^37^^,^^58^ Our results showed that the antagonist of SSTR1 decreased BMMSC migration toward cancer spheroids (Figure S9). As SST is known to be secreted by pancreatic cells,^59^ it may have been used as a cell attractant for the spheroids generated from the pancreatic cancer cell line in our experiment. Further studies are warranted to fully understand the properties of hMSCs as carrier cells.

Our study indicated that MSC origin influenced the cells’ susceptibility to oHSV (Figure 5) and thus may be a crucial factor in their functionality as viral carrier cells. MSCs express numerous surface receptors for some herpes virus genera and are susceptible to infection by HSV-1, varicella-zoster virus, and cytomegalovirus (CMV), although MSCs appear to be resistant to Epstein-Barr virus and human herpes virus infection.^60^ Moreover, MSCs are generally susceptible to HSV infection and express the HSV entry receptors nectin-1, herpesvirus entry mediator, and 3-O-sulfated heparan sulfate.^61^ Consistent with these previous findings, we found no significant differences in oHSV entry (Figure 5B). However, interestingly, our data suggested that the sensitivity of hMSCs to oHSV depended on their origin. For example, ADMSCs were comparatively resistant, whereas BMMSCs were vulnerable to oHSV infection (Figure 5A), which may be due to the release of oHSVs (Figure 5C). Furthermore, the efficiency of oHSV spread in cancer spheroids varied among the hMSCs (Figures 6 and 7). In the HSV release process, HSV nucleocapsids are enveloped by tegument-coated capsids in the cytoplasm of infected cells. The trafficking and release of HSV virions into the plasma membrane are regulated by host factors, including the dynein motor complex, kinesin motor protein, and Rab GTPases.^62^^,^^63^^,^^64^ A recent study reported that the cellular factor PTP1B is associated with the cell-to-cell spread of HSV.^65^ These mechanisms may be implicated in the susceptibility of hMSCs to oHSV. Therefore, the source of hMSCs should be carefully considered when using hMSCs in cancer gene therapy. Moreover, it has been reported that hMSC origin influences the effects of antitumor agents, including prodrugs,^66^^,^^67^ pro-apoptotic proteins,^68^^,^^69^ and growth factor antagonists.^70^^,^^71^ Identifying factors critical for hMSC-based cancer therapy will be useful for selecting appropriate hMSC sources and evaluating the functionality of hMSCs as oHSV carrier cells.

OVs have problems such as being neutralized by the host immune system and the difficulty of specific migration to the tumor site with systemic administration. Furthermore, OVs administered systemically in isolation are rapidly sequestered by the liver and spleen reticuloendothelial system, hindering their ability to reach the tumor *in vivo*.^72^ OVs delivered via hMSCs as carrier cells exert more potent antitumor effects than OVs alone.^62^ The primary advantage of using carrier cells is that they protect OVs from antiviral antibodies. Otherwise, they can invade the TME by reacting with tumor-secreted chemokines, potentially leading to their accumulation at the tumor site. Furthermore, immunosuppression by soluble factors from MSCs and direct cellular interactions of MSCs^72^^,^^73^^,^^74^ allows OVs not only to be protected from host immunosurveillance, but may also suppress local inflammation during virotherapy, thus allowing OVs to replicate and kill tumor cells without any immune restriction.^75^ In addition, MSCs loaded with OVs have the potential to act as virus-producing factories, which generate OV progeny at the tumor bed.^76^ On the other hand, MSC-based therapy faces challenges common to many cell therapies. For example, there is a possibility of an immune response or rejection, and the effects are also thought to vary between individuals. In addition, MSC-based therapy requires advanced technology and specialized facilities, making it costly. Furthermore, in stem cell therapy, stem cells may differentiate into undesirable lineages without limit, leading to tumor formation and inflammation.

Notably, our study showed that MSCs were rapidly killed by oHSV after infection in 2D culture and 3D co-culture (Figures 5A and S12), which limits monitoring of oHSV-MSC dynamics *in vitro*. Similarly to our observation *in vitro*, MSCs were killed by oHSV after infection within approximately 5 days *in vivo*,^77^ although MSCs reach the tumor within 4–24 h.^78^ While viral replication within MSCs is a desirable feature, excessive viral replication may result in premature MSC lysis and reduce overall efficiency.^79^^,^^80^ To address this issue, we intend to prevent OV replication during homing of oHSV-MSCs to tumor sites in future studies.

We established an efficient 3D co-culture model enabling sequential imaging of oHSV-hMSC movement and oHSV spread and antitumor effects. Using the 3D co-culture model, we demonstrated that particular hMSCs considerably improved the antitumor efficacy of oHSV (Figure 7). However, our 3D co-culture model leaves room for improvement. For instance, it does not allow determining the effects of the immune system and TME on oHSV-hMSCs. 3D co-culture models have been developed, including immune cells and CAFs that recapitulate the tumor immune microenvironment.^81^^,^^82^^,^^83^ By combining this approach with our culture model, cell-cell interaction among MSCs, immune cells, and CAFs during oHSV-hMSC therapy could be elucidated. Meanwhile, the 3D co-culture system in this study is flawed. An assay that would allow us to continue to observe the migration of oHSV-hMSCs to tumors and their effect on the tumors would be useful. However, our 3D co-culture model is artificial, and hMSCs infected with OVs typically only survive for a short period of time.^80^ When we performed a migration assay in a 3D co-culture model using oHSV-BMMSCs, the migration of oHSV-BMMSCs could not be monitored (Figure S12). To observe the dynamics and oncolytic activity of oHSV-hMSCs in 3D co-culture, we established a novel 3D co-culture model by pre-attachment of oHSV-hMSCs to the surface of cancer spheroids. Further improvement is required to evaluate the therapeutic efficacy of oHSV-hMSCs in various situations.

Various 3D culture systems have been developed, including suspension cultures in low-attachment plates, gel-like substances, or scaffolds.^84^ However, 3D culture systems have some obstacles. First, in low-attachment plates, cells spontaneously form large aggregates.^84^ Second, some materials are not conducive to single-cell migration. Thus, for the establishment of appropriate 3D culture systems, a range of conditions is needed to be considered encompassing cell density, scaffold, and culture method.^85^^,^^86^ To observe the 3D dynamics of hMSCs, we optimized the culture conditions regarding cell density, Matrigel concentration, and culture method. In our 3D co-culture model, the 3D dynamics between hMSCs/oHSV-hMSCs and cancer spheroids could be quantitively monitored, confirming the validity of our research. In addition, we also showed that our 3D co-culture model can be established with several types of cancer cell lines, and the migration ability of BMMSCs toward a spheroid can be monitored as well, suggesting that our 3D co-culture model is a versatile tool to test the migration ability of hMSCs for various types of cancer. Furthermore, by replacing cancer spheroids with primary tumor organoids from patients, our system could be used to evaluate the effectiveness of cancer gene therapies and establish personalized cancer gene therapies in the future.

In conclusion, our study revealed that MSCs have distinct characteristics as carrier cells depending on their tissue of origin, which highlights the significance of appropriately selecting the source of MSCs. Furthermore, we demonstrated that the antitumor effects of OV can be enhanced by employing MSCs as carrier cells. We provided experimental results obtained from a 3D co-culture model that more closely mimics the *in vivo* environment than 2D systems. These results suggested that the utilization of OV-MSCs may overcome the limitations of OV monotherapy and amplify the potency of OV-based therapeutic approaches.

## Materials and methods

### Ethics statement

The experimental protocols involving human participants were approved by the Ethics Committee of the Department of Medicine, Graduate School of Medicine, Nippon Medical School. All experiments involving materials derived from humans were performed following the guidelines of the Declaration of Helsinki and were approved by the Ethics Committee of Fukushima Medical University (Fukushima, Japan; approval nos. 1953 and 2192; approval dates March 18, 2020, and May 26, 2016, respectively). All participants provided written informed consent. All experimental procedures were performed according to guidelines approved by the Nippon Medical School Animal Ethics Committee.

### Cell culture

hMSCs and MIA PaCa-2/CMV-Luc (JCRB1681) were purchased from the Japanese Collection of Research Bioresources (JCRB, Tokyo, Japan) and were derived from bone marrow (BM; JCRB1151), adipose tissue (AD; JCRB1572), umbilical cord blood (UCB; JCRB1546), and endometrium (EPC; JCRB1536). Primary hMSCs derived from BM were acquired from Takara Bio (D1032, Shiga, Japan). Immortalized human fibroblasts (fHDF/TERT166, HDF; no. CHT-031-0166) were obtained from Evercyte (Vienna, Austria). Human bile duct cancer organoids (RBIL001) were procured at the Fukushima International Research Center (Fukushima, Japan). hMSCs were maintained in the basal medium of the Mesenchymal Stem Cell Growth Medium BulletKit culture system (PT-3001, Lonza, Walkersville, MD). Vero cells (CCL-81) and U2OS cells (HTB-96) were purchased from the American Type Culture Collection (ATCC, Manassas, VA). PANC-1, HDF, HEK293T, Vero, and U2OS cells were cultured in Dulbecco’s modified Eagle’s medium (DMEM) supplemented with 10% fetal bovine serum (FBS) and 1% penicillin/streptomycin (P/S). MIA PaCa-2/CMV-Luc and T24 cells were cultured in Eagle’s minimum essential medium with 10% FBS and 1% P/S. DLD-1 cells were cultured with RPMI-1640 with 10% FBS and 1% P/S. Bile duct cancer organoids were sustained in Cancer Cell Expansion Medium plus (032-25745, FUJIFILM Wako Pure Chemical, Osaka, Japan), as described previously.^87^ PANC-1 and HDF spheroids were generated as follows: 2 × 10^4^ cells were seeded in a 96-well U-bottom plate with a cell-repellent surface (650970, Greiner Bio-one, Frickenhausen, Germany) and centrifuged at 1,000 × *g* for 10 min. After 3 days of cultivation at 37°C, the spheroids were used for migration and cancer-killing assays.

### HF10-mCherry BAC construction

A targeting plasmid to insert bacterial artificial clone (BAC) into the UL3-4 locus of the HF10 genome was constructed as follows: the UL3 and 4 genomic fragments of HF10 were PCR-amplified using primers 1 and 2, and 3 and 4, respectively. A CMV-AcGFP fragment was amplified from pAcGFP-N1 (Takara Bio) using primers 5 and 6. All three fragments were recombined into pBluescript using the SacI and KpnI restriction enzyme sites to create pBS-UL3-4-CMV-AcGFP. pBS-UL3-4-linker was constructed by replacing the CMV-AcGFP cassette with a PacI linker that was PCR-amplified using primers 7 and 8. The EF1α promoter and mCherry gene were PCR-amplified using primers 9 and 10, and 11 and 12, respectively, and cloned into the SfoI-BamHI site of pBeloBAC (New England Biolabs) to create pBeloBAC-mCherry. pBeloBAC-mCherry was digested with PacI and cloned into the PacI site of pBS-UL3-4-linker (pBeloBac-UL3-4-mCherry). Finally, pBeloBAC-UL3-4-mCherry was linearized by PacI digestion and purified using Wizard SV Gel and PCR Clean-Up System (A9282, Promega, Madison, WI). U2OS cells were transfected with linearized pBeloBAC-UL3-4-mCherry and then infected with HF10, as described previously.^88^ When 100% of the cells showed a cytopathic effect (CPE), viral supernatants were harvested, and viral titers were measured using a standard plaque assay. mCherry-positive plaques were isolated by limiting dilution (HF10-mCherry BAC). HF10-mCherry viruses were expanded on Vero cells, and viral DNA was purified. The BAC sequence was verified through diagnostic digestion and sequence analysis. All primers used in this study were listed in Table S1.

### Viruses

oHSV strain HF10,^89^ Canerpaturev (C-REV, formerly known as HF10), obtained from Takara Bio, was grown in Vero cells. In brief, confluent Vero cells in T-225 culture flasks were infected with HF10 or HF10-mCherry at a multiplicity of infection (MOI) of 10^−3^ at 37°C for 2 h and then incubated at 33°C until 100% of the cells showed CPE. After treatment with 5 M NaCl at a volume of 10% of the medium at room temperature for 2 h, virus supernatants were harvested. Cell debris was removed through centrifugation at 1,110 × *g* (3,000 rpm) for 10 min at 4°C. Then, the samples were filtered through 0.8- and 0.45-μm membrane filters (Millex; Merck Millipore, Carrigtwohill, Ireland) and centrifuged at 39,900 × *g* (18,000 rpm) for 40 min to retrieve the virus. Physical and biological titers were determined as described previously.^90^

### FACS analysis

hMSCs were detached using TrypLE (12604013, Thermo Fisher Scientific, Waltham, MA). The cells were suspended in fluorescence-assisted cell sorting (FACS) buffer (2% FBS in PBS), and the suspensions were stained with the following antibodies: Brilliant Violet 421 anti-human CD73 antibody (no. 344008, BioLegend, San Diego, CA), YG-PE/Cy7 anti-human CD90 antibody (no. 328124, BioLegend), and APC anti-human CD105 antibody (no. 323208, BioLegend). Non-specific fluorescence was determined using isotype-matched mouse monoclonal antibodies (BioLegend). For the viability assay, hMSCs and PANC-1 cells were stained with Zombie NIR dye (no. 423105, BioLegend) at a 1:300 dilution for 20 min at room temperature. After washing once with FACS buffer, the viability was evaluated by flow cytometric analysis. FACS analysis was performed using a BD LSRFortessa X-20 cell analyzer (BD Biosciences, San Jose, CA). mCherry-positive PANC-1 cells and AcGFP-positive hMSCs were isolated using a BD FACSAria II system.

### Lentiviral vector construction

Lentiviral AcGFP expression plasmid (pCDH-EF1α-AcGFP-Bla) was constructed by cloning EF1α promoter and AcGFP cDNA, PCR-amplified using primers 13/14 and 15/16, respectively, into the SpeI/BamHI site of pCDH-CMV-MCS-SV40-blast (Miyagawa et al. PNAS). Lentiviral mCherry expression plasmid (pCDH-EF1α-mCherry-Bla) was constructed by cloning mCherry cDNA, PCR-amplified using primers 17/18 into the EcoRI/NheI site of pCDH-EF1α-AcGFP-Bla. Lentiviral vector production was performed as described previously.^91^ In brief, HEK293T cells were transfected with lentiviral plasmids and co-transfected with ViraPower Lentiviral Packaging Mix (Invitrogen, Carlsbad, CA) using PEI MAX at 37°C overnight. Then, the culture media were replaced with DMEM containing 1% P/S, and the cells were incubated at 33°C. Three days later, lentivirus supernatants were collected, centrifuged at 1,110 × *g* for 30 min, and filtered through a 0.45-μm filter. Finally, the virus was concentrated using LentiX concentrator (631232, Clontech, Palo Alto, CA) according to the manufacturer’s protocol.

### Lentiviral transduction

hMSCs were transduced with a lentivirus expressing AcGFP, and PANC-1 cells were transduced with a lentivirus expressing mCherry. A solution containing the lentiviral particles and polybrene (4 μg/mL) was added to the cell cultures. The cells were centrifuged at 800 × *g* for 30 min to increase the infection rate and cultured at 37°C. At 80% confluence, the transduced cells were selected using 5 μg/mL blasticidin.

### Determination of qRT-PCR-based viral titer and gene expression level of hMSCs

Viral physical titers (genome copies, GC) were determined as described previously.^90^ To evaluate the oHSV infection efficiency in hMSCs, 3 × 10^5^ hMSCs were infected with oHSVs at an MOI of 2 and subjected to rotation culture at 37°C for 1 h. The infected MSCs were collected by centrifugation at 1,000 rpm for 5 min at 4°C and washed three times with PBS. The cells were lysed with 0.1% NP-40, and cell nuclei were extracted as described previously.^92^ The oHSV GC in cell nuclei was determined as described previously.^90^ To examine the release of oHSVs from hMSCs, 1 × 10^4^ hMSCs were seeded in a 96-well plate. After 2 h of incubation at 37°C, the cells were infected with oHSVs at an MOI of 1 or 10. On day 2, supernatants were collected, and viral titers were determined through qPCR as described previously.^90^ To determine the SSTR1 gene expression level of hMSCs, hMSCs cultured in a 2D environment at 80% confluency were harvested. RNA was extracted from hMSCs using RNeasy Mini Kit (no. 74104, QIAGEN, Valencia, CA) according to the manufacturer’s protocol. Expression analysis of SSTR1 was performed by qRT-PCR using SSTR1 targeting primer 19 and 20. The data were normalized to cellular 18S rRNA as described previously.^93^ The gene expression level was calculated relative to the BMMSCs.

### 2D horizontal migration assay

The 2D horizontal migration assay was performed using two-well cell-culture inserts (no. 80209, Ibidi, Martinsried, Germany) in a 24-well plate. hMSCs (2 × 10^4^) were seeded onto one side of culture inserts and incubated at 37°C overnight. The following day, bile duct cancer organoids or HDFs (4 × 10^4^ cells) were suspended in 30 μL of Matrigel (356230, Corning, Corning, NY) and seeded on the other side of the culture inserts. Cell numbers in bile duct cancer organoids were visually estimated as described previously.^94^ The Matrigel was allowed to solidify at 37°C for 30 min. The cell-culture inserts were gently removed, the cells were washed once with PBS, and 500 μL of the basal medium of the Mesenchymal Stem Cell Growth Medium BulletKit culture system without FBS was added. The cells were incubated at 37°C for 72 h to monitor hMSC migration. Fluorescence micrographs of hMSCs were captured daily using a fluorescence microscope. The area of migrated hMSCs was measured using the Image-PRO 10 software (Hakuto, Tokyo, Japan).

### Vertical migration assay

The vertical migration assay was performed using Transwell chambers with 8-mm-pore polycarbonate filter inserts (353097, Corning) in a 24-well plate. PANC-CM was used as a chemoattractant and HDF-CM worked as a negative control, as described previously.^95^ hMSCs (5 × 10^4^) were seeded on the inserts, and the lower chambers were filled with 500 μL of PANC-CM or HDF-CM. The hMSCs were allowed to migrate at 37°C for 24 h and then stained with Hoechst 33342 (R37605, Thermo Fisher Scientific). Stained cells were counted in five random high-power fields per chamber using a fluorescence microscope.

### 3D migration assay

hMSCs (5 × 10^3^) or oHSV-BMMSCs (5 × 10^3^) were seeded in the basal medium of the Mesenchymal Stem Cell Growth Medium BulletKit culture system without FBS containing 5% Matrigel in a low-attachment 96-well plate (3474, Corning). A PANC-1, DLD-1, T24, U2OS, or HDF spheroid was placed carefully in the center of each well. For the assay with SSTR1 antagonist, CYN154806 (37343, Cayman) was added to the culture medium at 0.1, 0.5, and 4.0 μM. Confocal z stack images (step size 3 μm, 55× slices, 40× objective) were captured daily using a confocal microscope (FV1200, Olympus, Tokyo, Japan). 3D images were analyzed, and the distance between the hMSCs and spheroids was determined using the Image-PRO software.

### RNA sequencing analysis

hMSCs in 3D migration assays were collected via FACS using a FACSAria Fusion cell sorter (BD Biosciences). The cells were lysed using single-cell lysis buffer (Takara Bio) supplemented with 60 units of RNaseOUT (Invitrogen). Total RNA was purified using RNAClean XP beads (Beckman Coulter, Brea, CA). RNA sequencing libraries were generated using an SMART-seq HT Plus Kit (Takara Bio) according to the manufacturer’s instructions. Sequencing was performed on a HiSeqX (Illumina, San Diego, CA) platform, generating 2 × 150-bp reads. After removing the adapter sequences and low-quality reads using fastp, paired-end reads were mapped to the mm10 genome using STAR with default parameters. Transcripts were quantified using analyzeRepeats in HOMER, with the parameters -condenseGenes -count exons -noadj.^96^ Transcript expression values were calculated using the analyzeRepeats.pl tool in HOMER, with the parameters -condenseGenes -count exons -tpm. DEGs were assessed and identified using DESeq2 based on an adjusted *p* value < 0.05 and fold change > 2.^97^

### Cytotoxicity assay

The cytotoxicity of oHSV toward the hMSCs was determined using the MTT assay.^90^ hMSCs (1 × 10^4^) were seeded in a 96-well plate. After 2 h, the cells were infected with oHSVs at an MOI of 0.03, 0.3, or 3. On days 2, 4, 6, and 8, viable hMSCs were assessed using the MTT assay as described previously.^90^

### Cell-killing assay

To determine the cell-killing ability of oHSVs using 2D culture, 5 × 10^3^ hMSCs infected or not with oHSVs expressing mCherry at an MOI of 2 for 2 h (oHSV-hMSCs) were seeded on a monolayer of PANC-1 cells (seeded at 4 × 10^4^ cells). As a control, PANC-1 cells were infected with an equal amount of oHSV in oHSV-hMSCs. Fluorescence micrographs were captured daily, and the spread rate was calculated using the Image-PRO software. Cell viability was determined using Cell Counting Kit-8 (CCK-8) (343-07623, Dojindo, Kumamoto, Japan) according to the manufacturer’s instructions. To determine the cell-killing ability of oHSVs using 3D culture, five non-labeled PANC-1 spheroids were infected with equal amounts of mCherry-expressing oHSV-hMSCs or co-cultured with 2 × 10^4^ oHSV-hMSCs in rotation culture at 36 rpm for 2 h at 37°C, as described previously.^43^ After the rotation culture, the PANC-1 spheroids were gently harvested, washed with fresh culture medium three times, and plated in a 96-well low-attachment plate. oHSV spread into the PANC-1 spheroids was imaged daily, and the spread rate was calculated using the Image-PRO software. On day 7, cell viability in the 3D culture was determined using the CCK-8.

### *In vivo* imaging of hMSC migration assay

*In vivo* imaging was carried out by using the Xenogen In Vivo Imaging System (IVIS) (Caliper Life Sciences, Hopkinton, MA). MIA PaCa-2/CMV-Luc pancreatic cancer xenografts were established by injecting 1 × 10^7^ MIA PaCa-2/CMV-Luc cells suspended in 300 μL of PBS into the abdominal cavity of 5-week-old female nude mice (BALB/cAJcl-nu/nu, *n* = 5). Three weeks after the inoculation, the tumor growth was assessed by measuring the luciferase activity using bioluminescence imaging (BLI). The mice were then injected peritoneally with 1 × 10^6^ BMMSCs or EPCMSCs labeled with IVISense DiR 750 Fluorescent Cell Labeling Dye (DiR) as described previously.^98^ The *in vivo* migration of hMSCs was visualized by BLI. Four days after hMSC injection, each mouse was injected with D-luciferin at 150 mg/kg body weight (100 μL) via intraperitoneal injection 5 min before imaging. Bioluminescence and fluorescence images of abdomen or excised tumors were analyzed using Living Image software (PerkinElmer) by manually defining the regions of tumors. Imaging data were normalized and expressed as radiance (p/s/cm^2^/sr) for bioluminescence or radiant efficiency ([p/s/cm^2^/sr]/[μW/cm^2^]) for fluorescence, and the color scale was adjusted according to the strength of signal.

### Statistical analysis

All statistical analyses were conducted using EZR (Saitama Medical Center, Jichi Medical University, Saitama, Japan, version 1.60), which is a graphical user interface for R (The R Foundation for Statistical Computing, Vienna, Austria, version 4.2.1).^99^ Specifically, EZR is a modified version of R Commander (version 2.8-0) that incorporates commonly used statistical functions in biostatistics. Student’s t test was employed to compare variables between the two groups. To analyze differences among more than three groups, the one-way analysis of variance (ANOVA) followed by Tukey’s multiple comparisons test was utilized. Statistical significance was determined at *p* < 0.05.

## Data and code availability

The datasets analyzed during this study are available from the corresponding authors upon reasonable request.

## Acknowledgments

We are grateful to Takenori Fujii (Nippon Medical School) for pathological analysis, Masumi Shimizu (Nippon Medical School) for FACS analysis, Takeshi Kijima (Yokohama General Hospital) for advice on 3D culture, and Yuka Ohyama and Izumi Yoshida (Nippon Medical School) for cell culture. We thank Takara Bio Inc. for providing C-REV. This research was supported by 10.13039/501100001691Japan Society for the Promotion of Science (21H03828) and 10.13039/100009665Vehicle Racing Commemorative Foundation (6225).

## Author contributions

M.S. and Y.M. conceived and designed the study. M.S., Y.M., S.K., Y.Y., M.Y., K.A., and Y.S. performed the experiments. M.S. and Y.M. analyzed the data. M.S. and Y.M. wrote the manuscript. Y.Y. conducted the animal experiments. Y.M., N.T., H.Y., A.U., M.S., and T.O. supervised the study. All authors reviewed and edited the manuscript.

## Declaration of interests

The authors declare no competing interests.

## Supplemental information

Document S1. Figures S1–S14 and Table S1

Document S2. Article plus supplemental information
