# Supplementary material for: Mesenchymal stem cell origin contributes to the antitumor effect of oncolytic virus carriers
Source: Mol Ther Oncol. 2024 Oct 18;32(4):200896. doi: 10.1016/j.omton.2024.200896 (PMC11568361; doi:10.1016/j.omton.2024.200896)
Supplement: Document S1. Figures S1–S14 and Table S1 [file mmc1.pdf]

OMTON, Volume *32*

Supplemental information

Mesenchymal stem cell origin contributes to the antitumor effect of oncolytic virus carriers

Makoto Sukegawa, Yoshitaka Miyagawa, Seiji Kuroda, Yoshiyuki Yamazaki, Motoko Yamamoto, Kumi Adachi, Hirofumi Sato, Yuriko Sato, Nobuhiko Taniai, Hiroshi Yoshida, Akihiro Umezawa, Mashito Sakai, and Takashi Okada

A

B

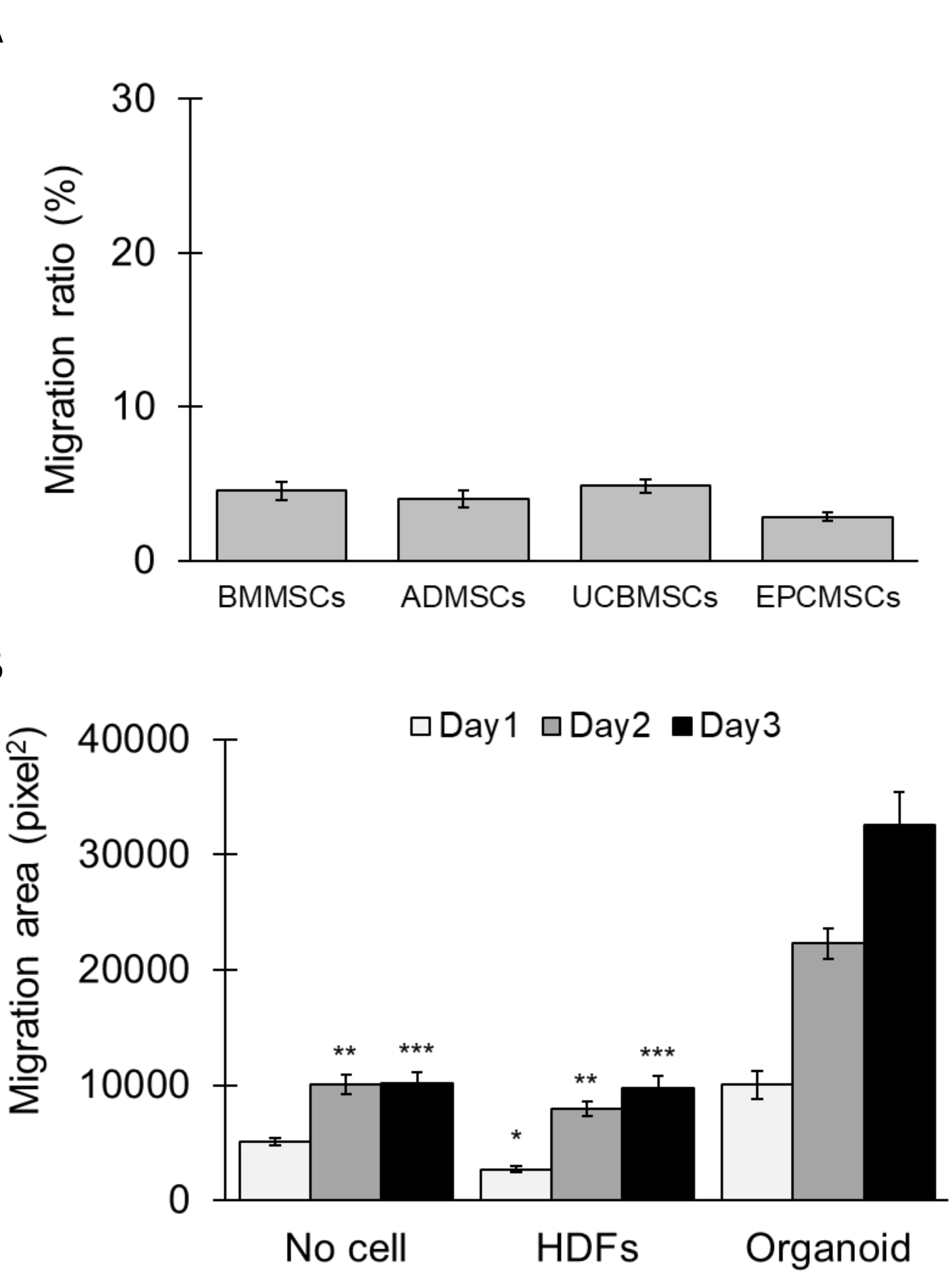

Figure S1. Evaluation of human mesenchymal stem cell (hMSC) tropism for non-cancerous cells in two-dimensional (2D) culture models.

(A) Migration ratio of hMSCs towards conditioned media of human dermal fibroblasts (HDF-CM) as determined using a Transwell vertical migration assay. **(B)** Migration efficiency of bone marrow-derived mesenchymal stem cells (BMMSCs) towards Matrigel without cells, human dermal fibroblasts (HDFs), or cancer organoids. The area occupied by migrated hMSCs was measured using the Image-PRO image analysis software (Hakuto). The assay was conducted in triplicate. Values are expressed as mean ± standard deviation (SD) (*, **, ***p < 0.05, one-way ANOVA followed by Tukey's multiple-comparison tests).

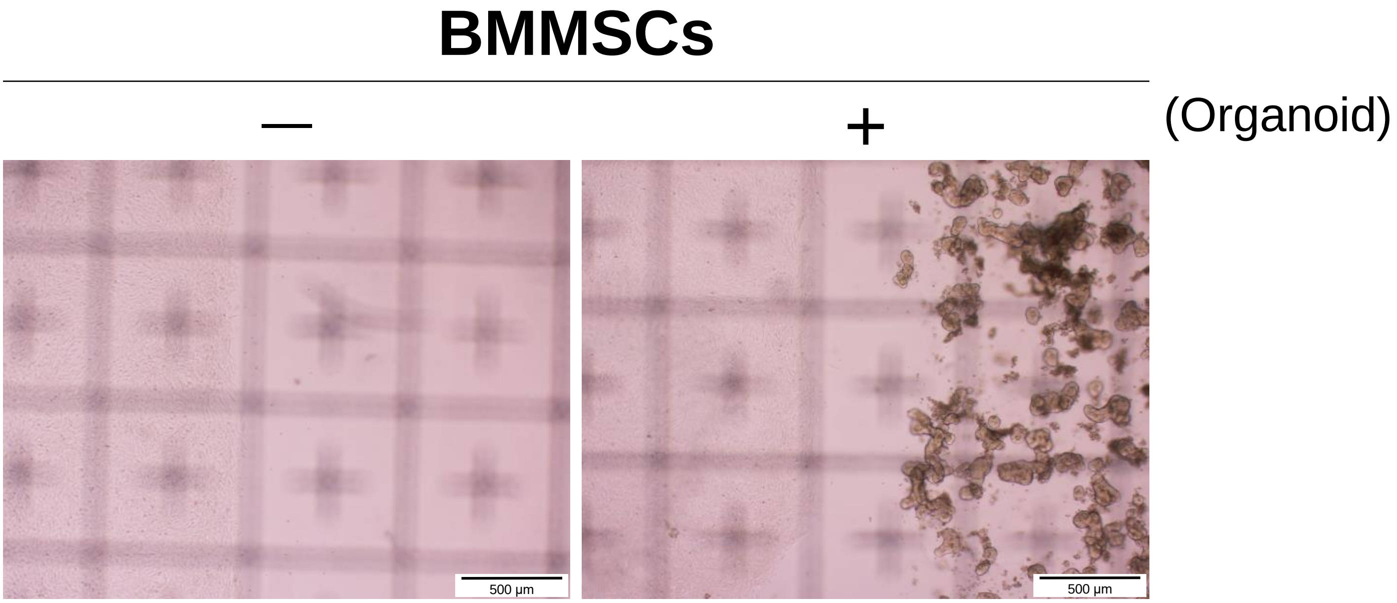

Figure S2. Representative bright-field microscopic images of the horizontal migration assay.

Migration assay of BMMSCs towards bile duct cancer organoids in 2D culture. BMMSCs and organoids were seeded in culture inserts that were placed in the wells of a culture plate. BMMSCs were seeded with (right) or without (left) organoids. The micrographs were captured on day 0. Scale bar represents 500 μm.

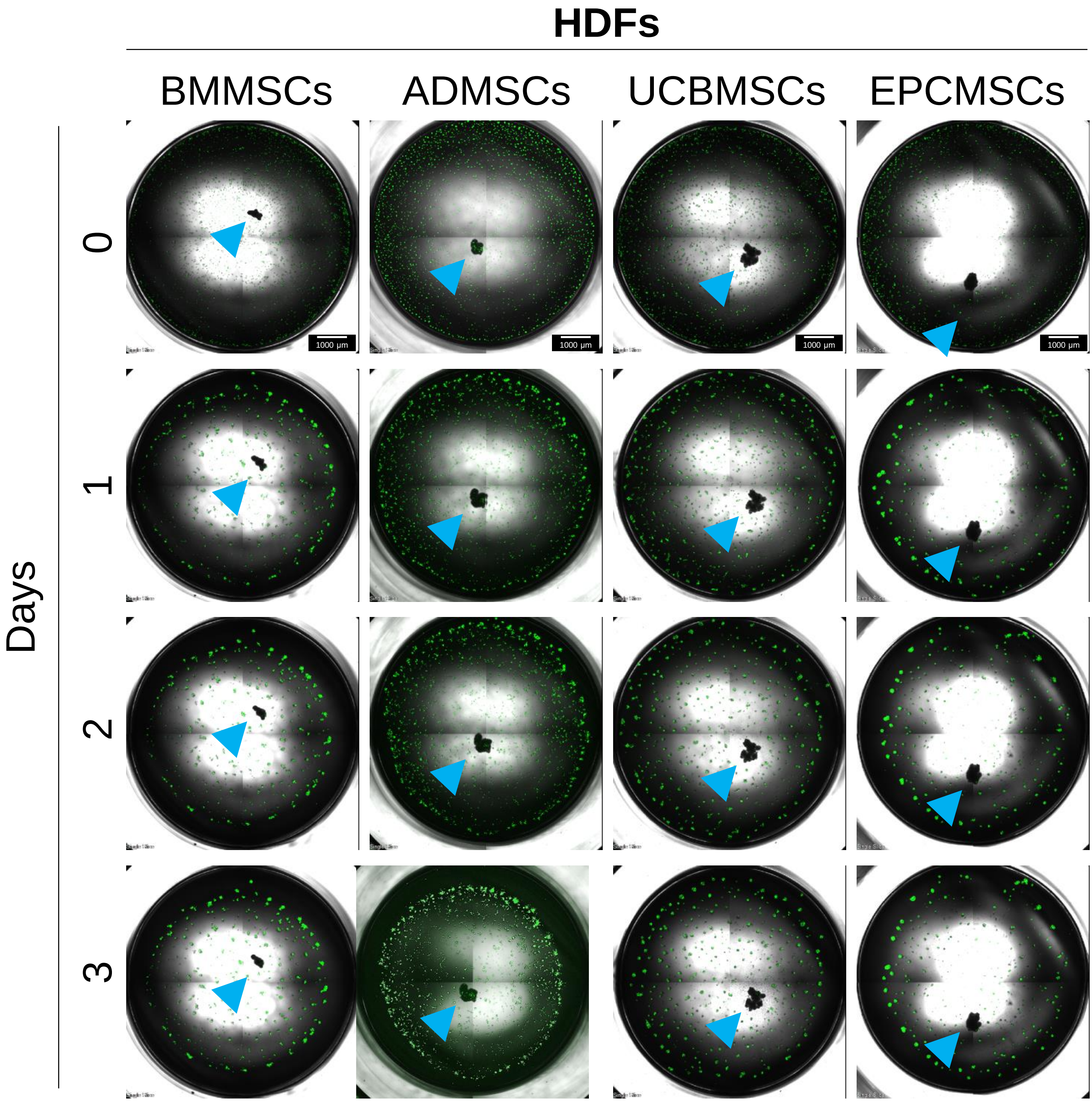

Figure S3. Bright-field microscopic images of the three-dimensional (3D) migration assay using HDF spheroids.

Monitoring of hMSC migration towards a HDF spheroid in 3D culture. hMSCs were suspended in serum-free medium containing Matrigel and seeded in a low attachment plate. Then, a HDF spheroid was placed in the Matrigel at the center of the well. Confocal z-stack images (step size 3 μm, 55 slices, 40× objective) were acquired daily using a confocal microscope (FV1200; Olympus). The micrographs are shown as maximum-intensity projections. hMSCs: green, HDF spheroids: not labeled. Blue arrowheads indicate HDF spheroids. Scale bar represents 1000 μm.

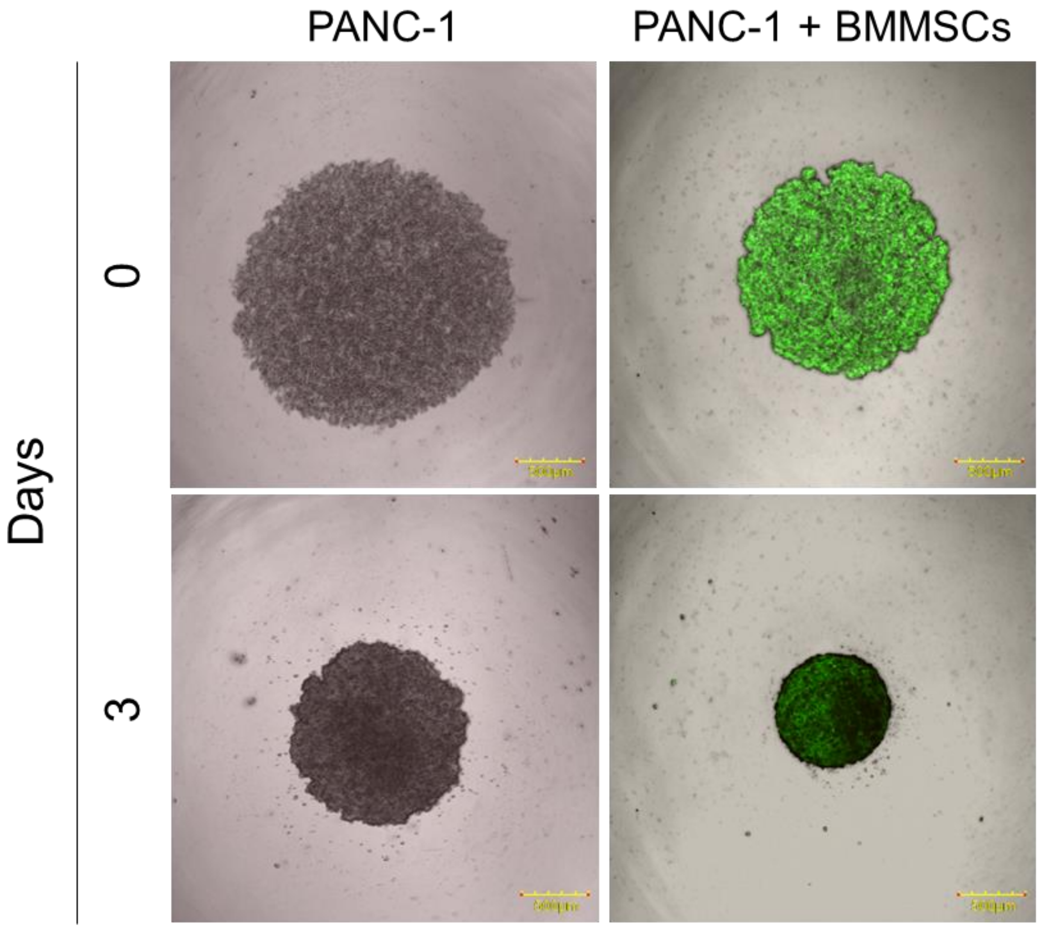

Figure S4. Morphology of a PANC-1 spheroid in 3D culture.
Micrographs of a spheroid formed from PANC-1 cells or PANC-1 cells and BMMSCs on days 0 and 3. PANC-1 cells: not labeled, BMMSCs: green. Scale bar represents 500 μm.

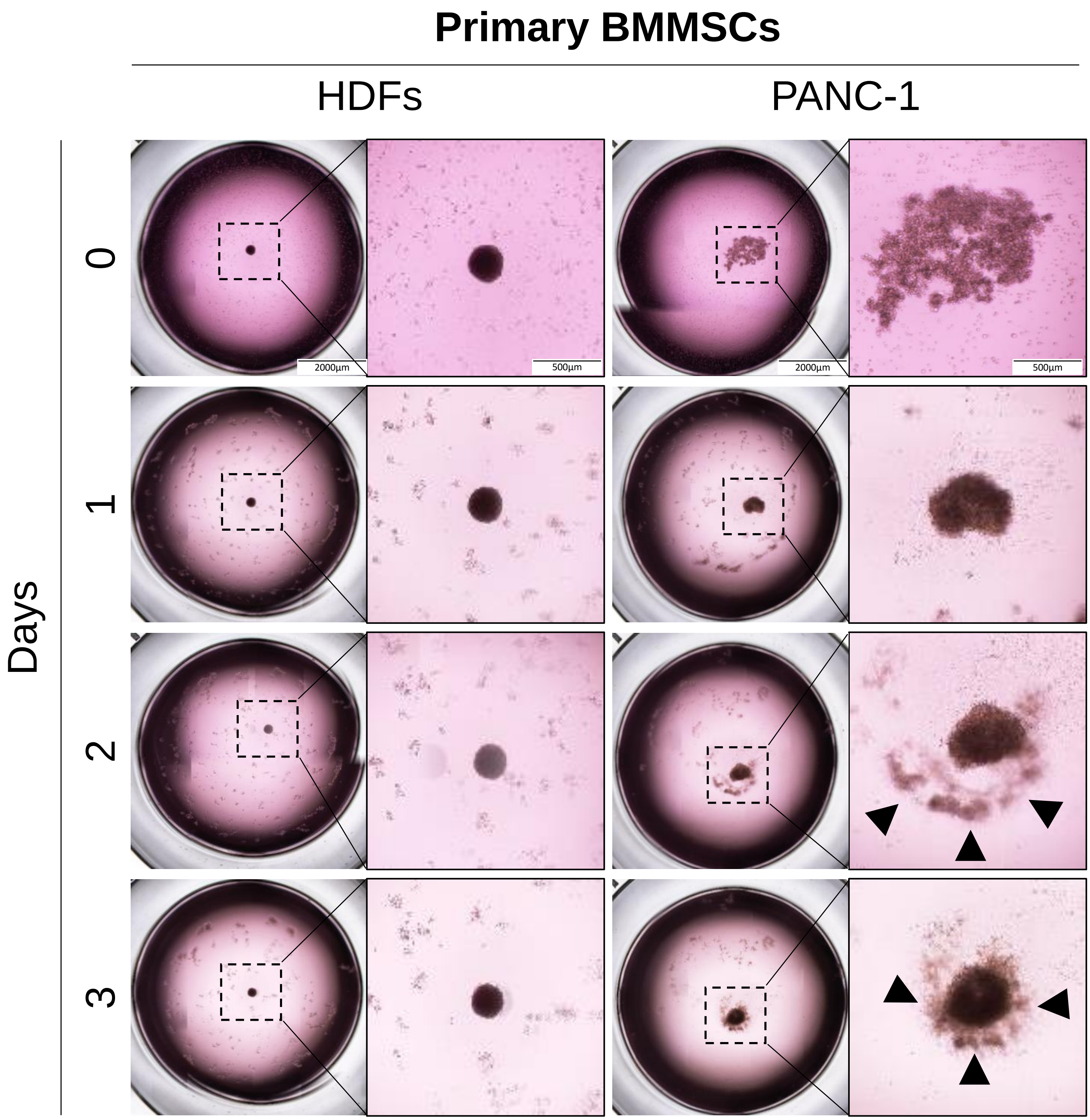

Figure S5. Primary BMMSCs exhibit tumor-specific tropism in a 3D culture system. Micrographs of 3D migration assays using primary BMMSCs and a HDF (left column) or PANC-1 (right column) spheroid. Primary BMMSCs were co-cultured with a spheroid in Matrigel in a low attachment plate. Images were acquired daily using a bright-field microscope. Scale bar represents 2000 μm. Magnified images of the areas around the spheroid are also shown. Scale bar represents 500 μm. Black arrowheads indicate BMMSCs migrating towards a PANC-1 spheroid.

A

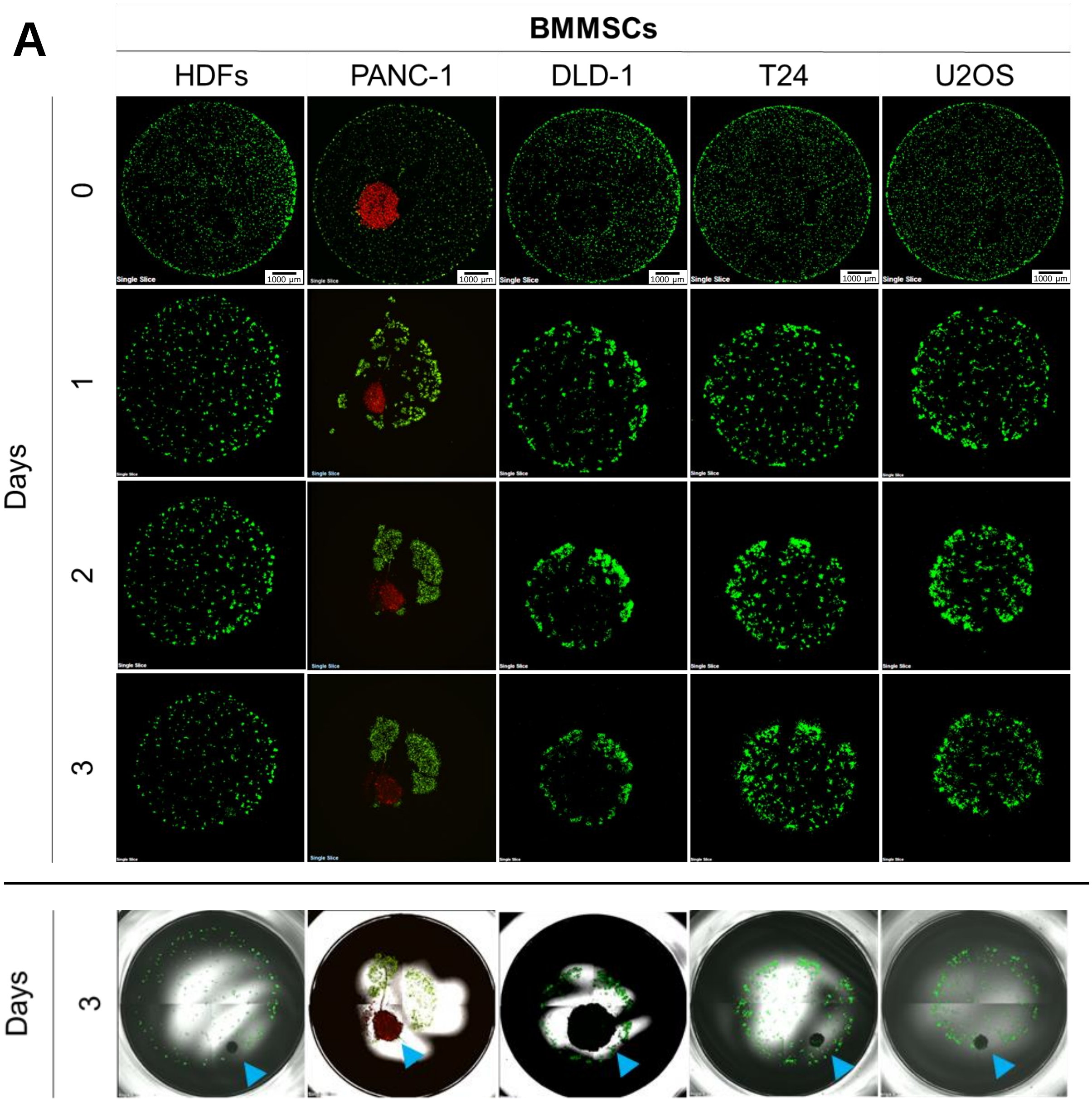
BMMSCs
HDFs
PANC-1
DLD-1
T24
U2OS
Days
0
1
2
3
1000 µm
Single Slice

B

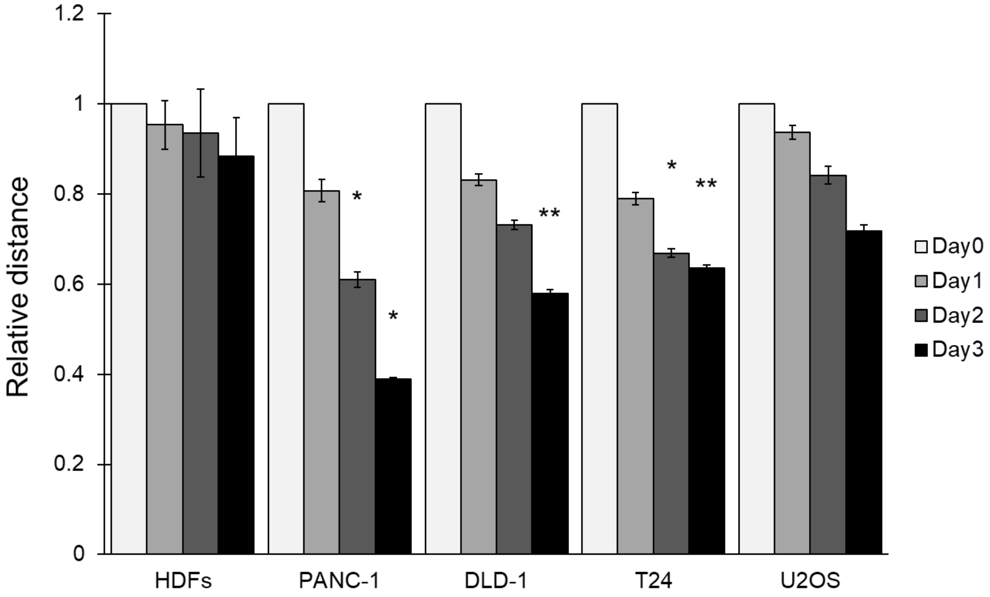
1.2
1
0.8
0.6
0.4
0.2
0
Relative distance
HDFs
PANC-1
DLD-1
T24
U2OS
Day0
Day1
Day2
Day3
*
**

Figure S6. BMMSC migration towards various types of cancer cells in a 3D co-culture model.

(A) Monitoring of BMMSC migration towards various spheroids in a 3D co-culture model. BMMSCs and spheroids derived from various cancer cell lines or HDFs were co-cultured in a low attachment plate. Confocal micrographs were captured daily and are shown as maximum-intensity projections. Bright-field images of non-labeled spheroids are also shown. BMMSCs: green, PANC-1 spheroid: red, HDF spheroid: not labeled, DLD-1 spheroid: not labeled, T24 spheroid: not labeled, U2OS spheroid: not labeled. Blue arrowheads indicate spheroids. Scale bar represents 1000 μm. **(B)** Migration ratio of BMMSCs towards spheroids in 3D co-culture. The migration ratio was calculated relative to the distance on day 0. The assay was conducted in triplicate. Values are expressed as mean ± SD (*, ***p* < 0.05 on days 2 and 3, one-way ANOVA followed by Tukey's multiple-comparisons test).

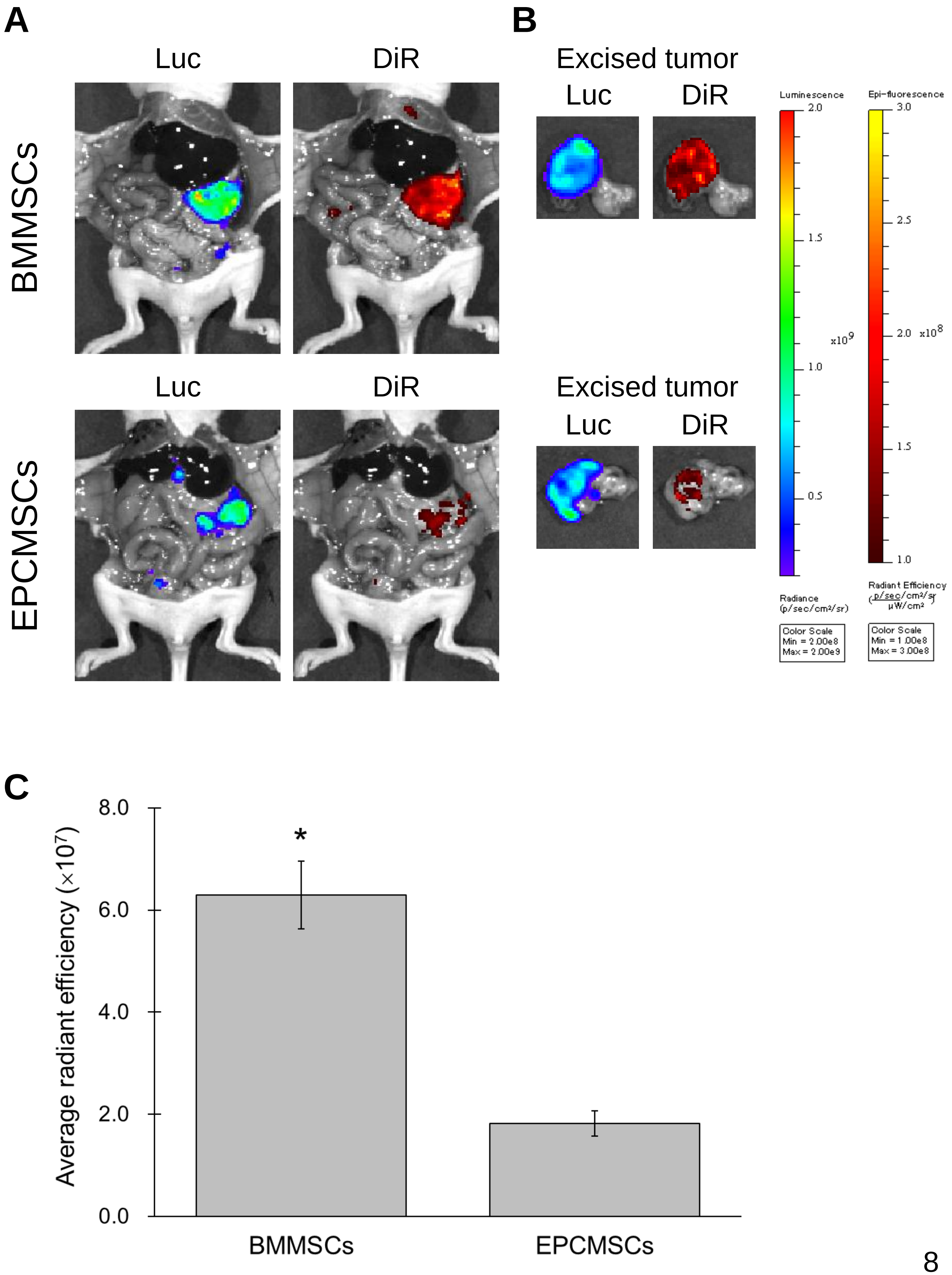
A
Luc
DiR
BMMSCs
Luc
DiR
EPCMSCs
B
Excised tumor
Luc
DiR
Excised tumor
Luc
DiR
Luminescence
2.0
1.5
1.0
0.5
x10^9
Radiance
(p/sec/cm²/sr)
Color Scale
Min = 2.00e8
Max = 2.00e9
Epi-fluorescence
3.0
2.5
2.0
1.5
1.0
x10^8
Radiant Efficiency
(p/sec/cm²/sr / µW/cm²)
Color Scale
Min = 1.00e8
Max = 3.00e8
C
Average radiant efficiency (×10^7)
8.0
6.0
4.0
2.0
0.0
*
BMMSCs
EPCMSCs

Figure S7. ***In vivo*** **and** ***ex vivo*** **Bioluminescence imaging (BLI) to monitor the hMSC migration toward cancer cells.**

BLI of MIA PaCa-2/CMV-Luc cells and hMSCs labeled with IVISense DiR 750 Fluorescent Cell Labeling Dye (DiR). MIA PaCa-2/CMV-Luc xenograft models were established by peritoneally injection of 1×10^7 MIA PaCa-2/CMV-Luc cells into 5-week-old female nude mice. Three weeks after the injection, 1×10^6 BMMSCs or EPCMSCs labeled with DiR were injected into the abdominal cavity of the xenografts. Four days after injection of hMSCs, bioluminescence and fluorescence signals were quantitatively measured by IVIS. BLI of MIA PaCa-2/CMV-Luc and BMMSCs or EPCMSCs *in vivo* **(A)** and *ex vivo* **(B)**. The xenografts were sacrificed, and the abdomen and excised tumors were measured the bioluminescence and fluorescence signals by IVIS. Optical images of the tumor and hMSCs obtained from IVIS were analyzed with Living Image Software. **(C)** *Ex vivo* quantitative BLI of migrated hMSCs toward cancer cells. Luc stood for MIA PaCa-2/CMV-Luc cells, and DiR stood for hMSCs labeled with IVISense DiR 750 Fluorescent Cell Labeling Dye in this figure. Values are expressed as the mean ± SD (n = 5, $*p < 0.05$ by Student's *t*-test).

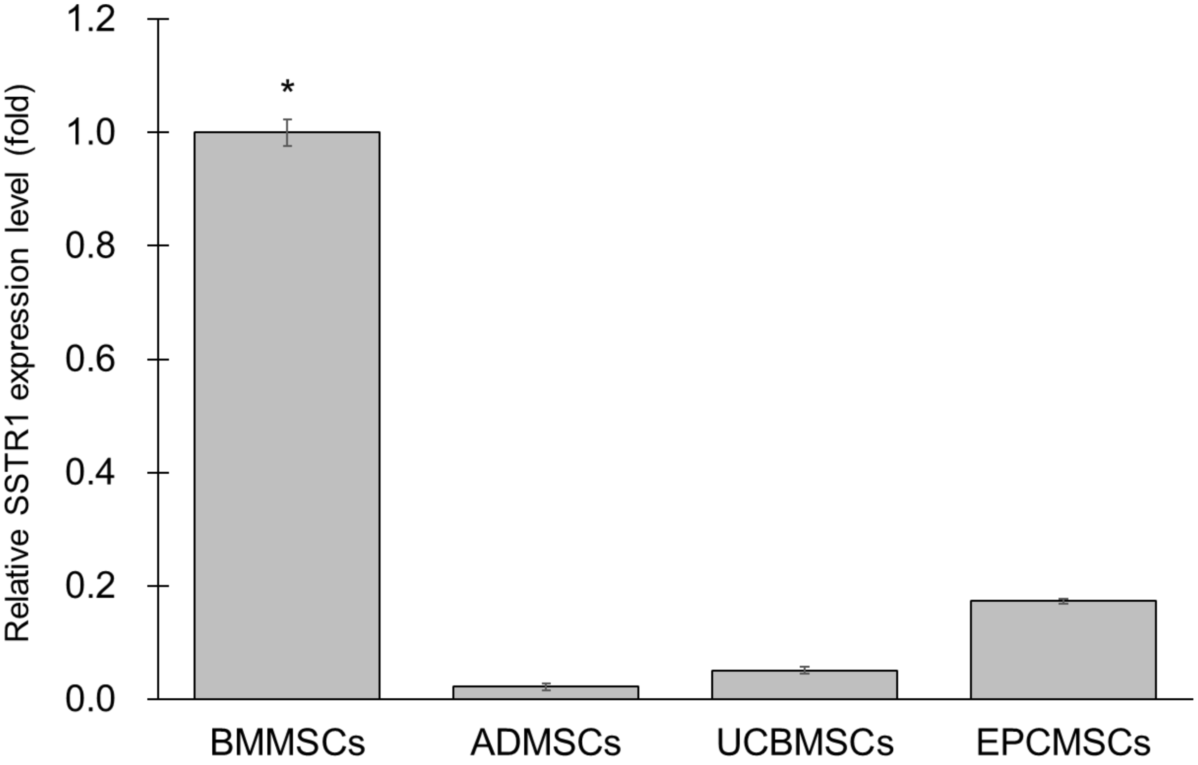

Figure S8. SSTR1 expression level in hMSCs.

SSTR1 mRNA levels in hMSCs as measured using reverse transcription quantitative PCR (qRT-PCR). hMSCs were harvested at 80% confluency. RNA was extracted, and *SSTR1* expression was analyzed using qRT-PCR. Target gene expression was normalized to that of 18S rRNA and calculated relative to that in BMMSCs. The assay was conducted in triplicate. Values are expressed as mean ± SD (*$p < 0.05$, one-way ANOVA followed by Tukey's multiple-comparisons test).

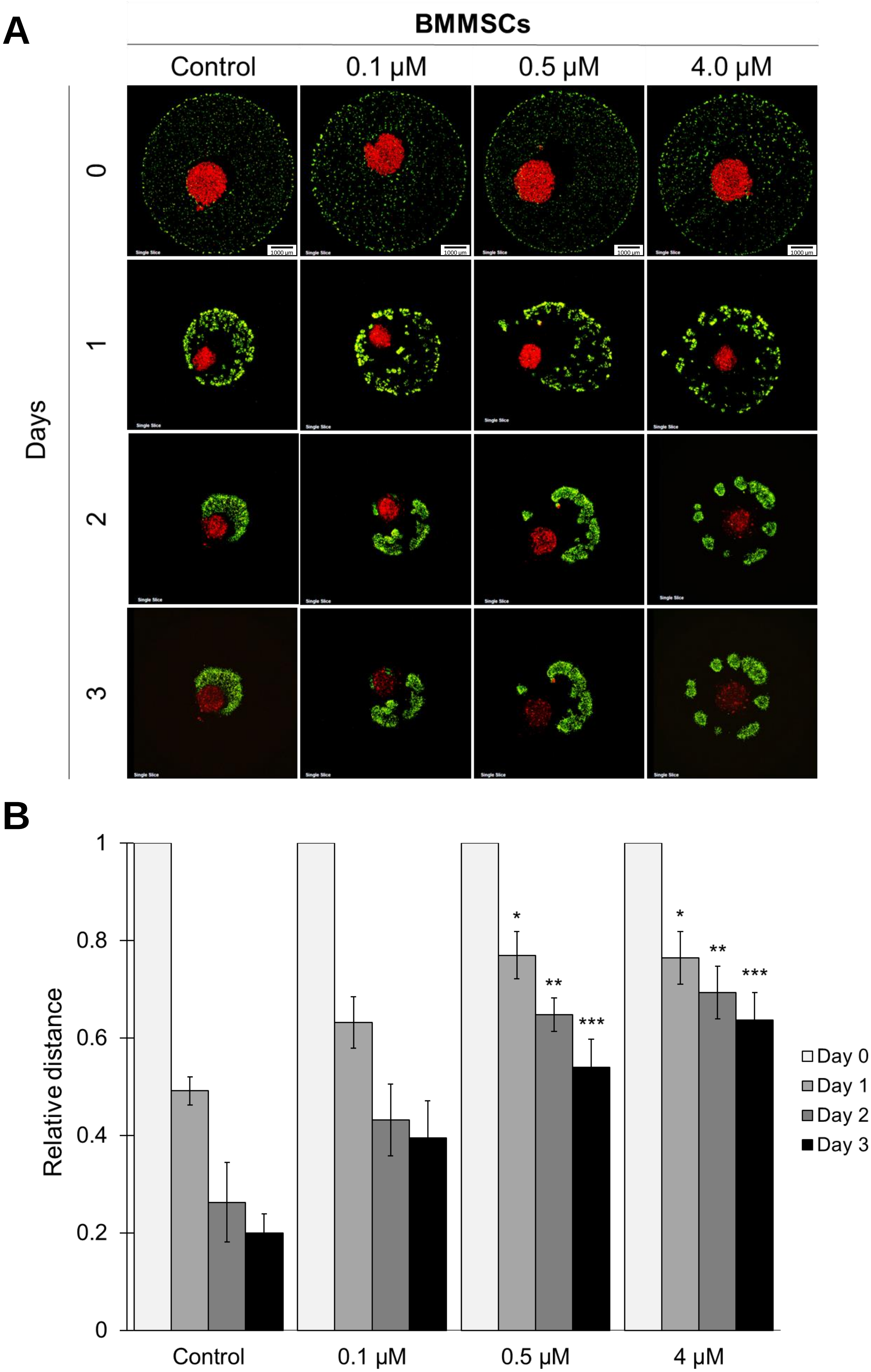
A
BMMSCs
Control
0.1 μM
0.5 μM
4.0 μM
Days
0
1
2
3
B
Relative distance
1
0.8
0.6
0.4
0.2
0
*
**
***
*
**
***
Day 0
Day 1
Day 2
Day 3
Control
0.1 μM
0.5 μM
4 μM

Figure S9. Effect of an SSTR1 antagonist on BMMSC migration towards cancer cells in a 3D co-culture model.

(A) Micrographs of BMMSC migration towards a PANC-1 spheroid in a 3D co-culture model in the presence of the SSTR1 antagonist CYN154806. The antagonist was added to the culture medium at a concentration of 0.1, 0.5, or 4.0 µM. Confocal micrographs were captured daily and are shown as maximum-intensity projections. BMMSCs: green, PANC-1 spheroids: red. Scale bar represents 1000 µm. **(B)** Migration ratio of BMMSCs towards a PANC-1 spheroid in 3D co-culture. The migration ratio was calculated relative to the distance on day 0. The assay was conducted in triplicate. Values are expressed as mean ± SD (*, **, ***$p < 0.05$, one-way ANOVA followed by Tukey's multiple-comparison tests).

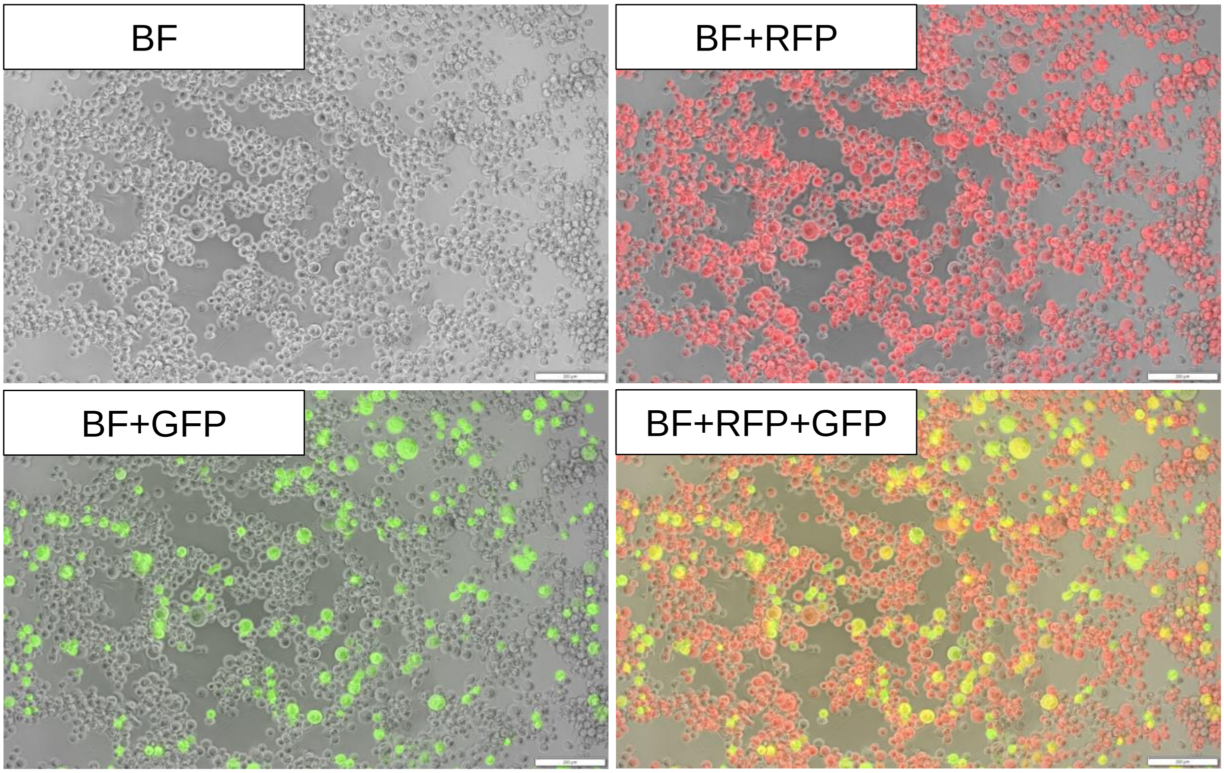

Figure S10. Spread of oHSVs in PANC-1 cells in a 2D co-culture model.
Micrographs of oHSV spreading in PANC-1 cells in a 2D co-culture model on day 2. PANC-1 cells were co-cultured with oHSV-mCherry-loaded hMSCs, and the spread of oHSV was monitored on day 2 using fluorescence microscopy. BF represents bright-field. PANC-1 cells: not labeled, BMMSCs: green, oHSV: red. Scale bar represents 200 μm.

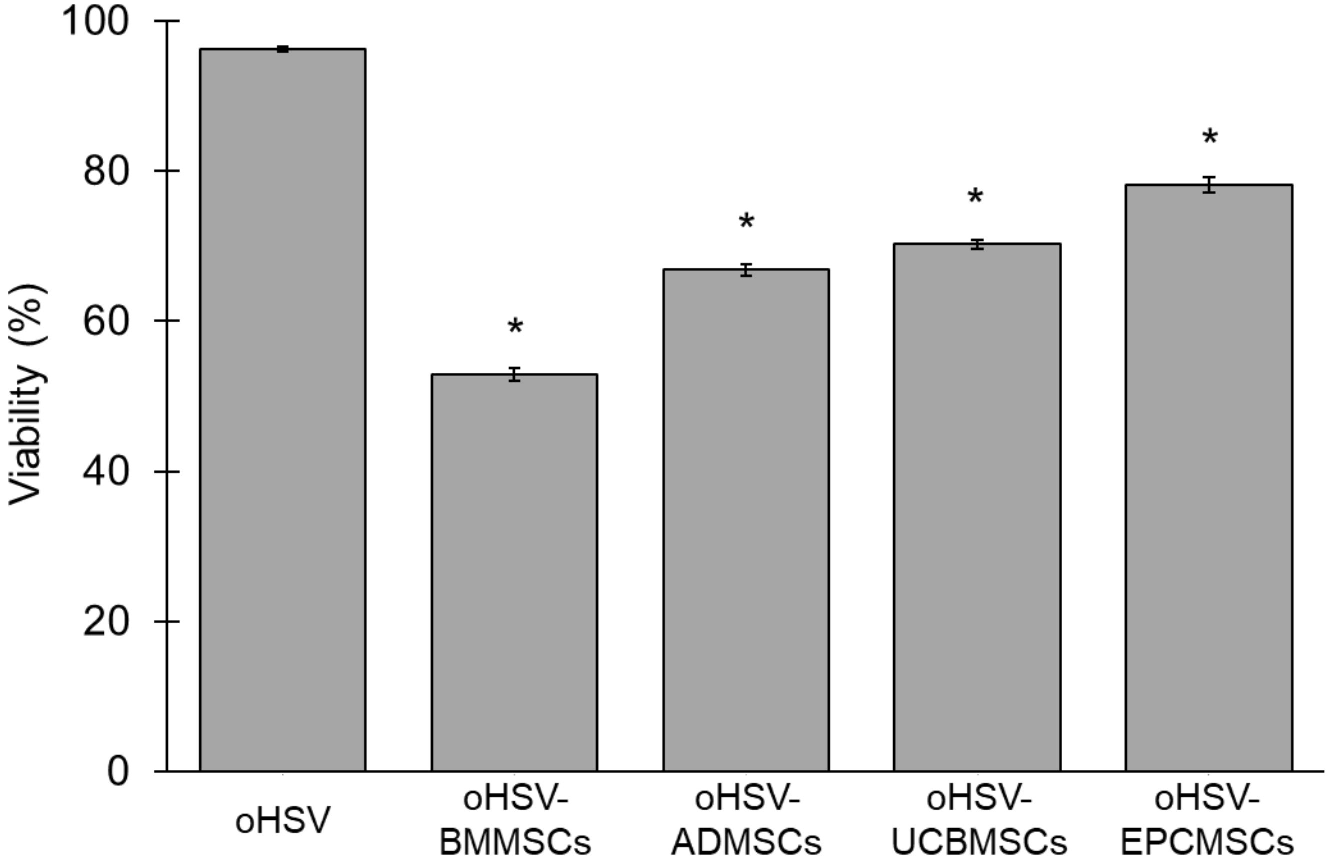

Figure S11. Viability of PANC-1 cells infected with oHSVs or co-cultured with oHSV-hMSCs in a 2D culture model.

PANC-1 cells were infected with oHSV or co-cultured with oHSV-hMSCs in 2D culture. Cell viability was determined using flow cytometry on day 2 after infection. GFP-negative cells were considered PANC-1 cells, and cell viability was analyzed by flow cytometry after staining dead cells with Zombie Aqua viability dye. The assay was conducted in triplicate. Values are expressed as mean ± SD (**p* < 0.05, one-way ANOVA followed by Tukey's multiple-comparisons test).

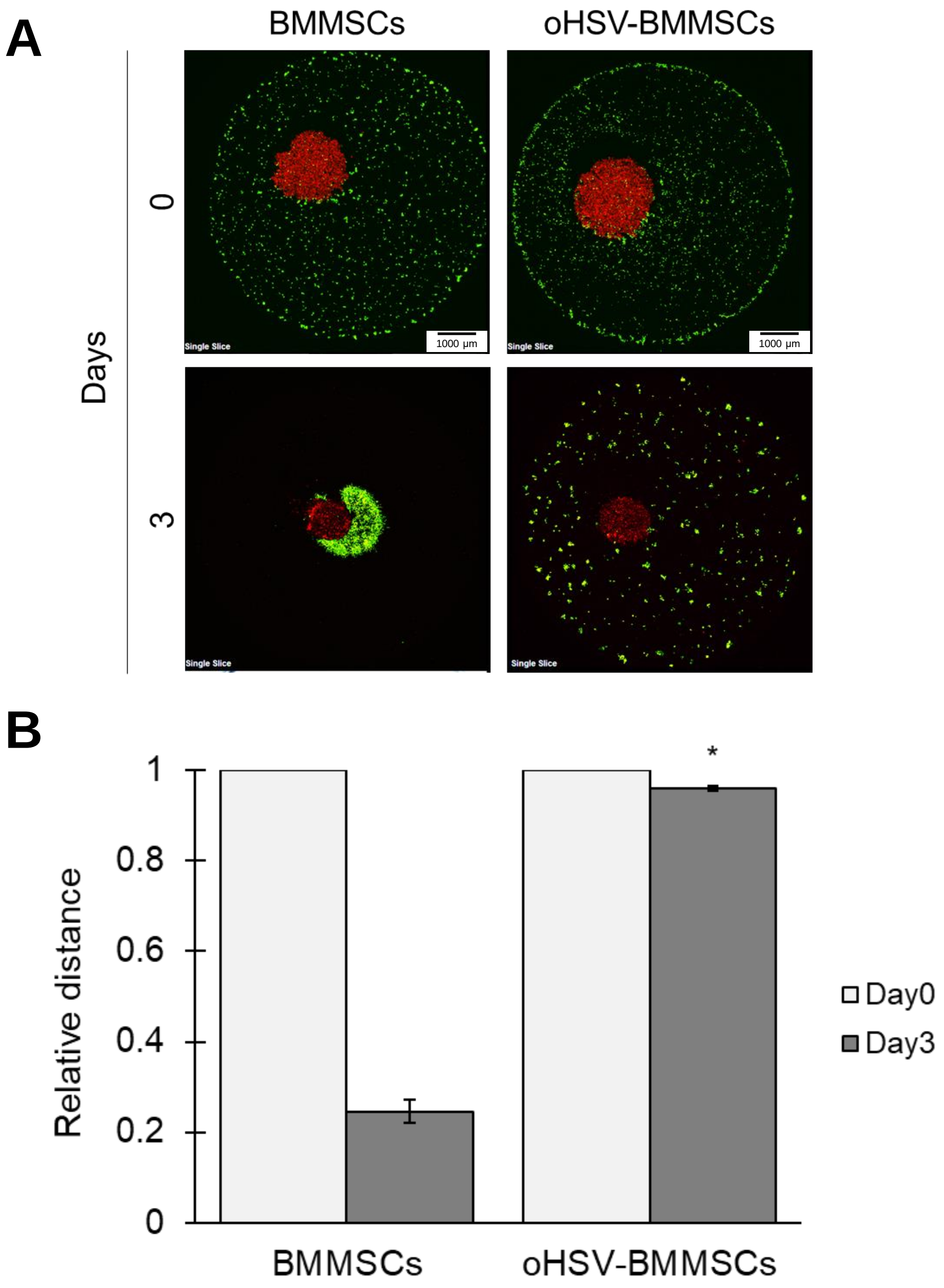

Figure S12. Evaluation of oHSV-BMMSC migration towards a PANC-1 spheroid in a 3D co-culture model.

(A) Micrographs of BMMSC and oHSV-BMMSC migration towards a PANC-1 spheroid in a 3D co-culture model. BMMSCs were infected with oHSV (oHSV-BMMSCs) at a multiplicity of infection (MOI) of 0.5 for 2 h. BMMSCs or oHSV-BMMSCs were co-cultured with a PANC-1 spheroid in a low attachment plate. The migration of BMMSCs or oHSV-BMMSCs was monitored using confocal microscopy. BMMSCs and oHSV-BMMSCs: green, PANC-1 spheroid: red. Scale bar represents 2000 μm. **(B)** Migration ratio of BMMSCs towards a PANC-1 spheroid in 3D co-culture. The migration ratio was calculated relative to the distance on day 0. The assay was conducted in triplicate. Values are expressed as mean ± SD (**p* < 0.05, Student's *t*-test).

PANC-1 spheroids with hMSCs

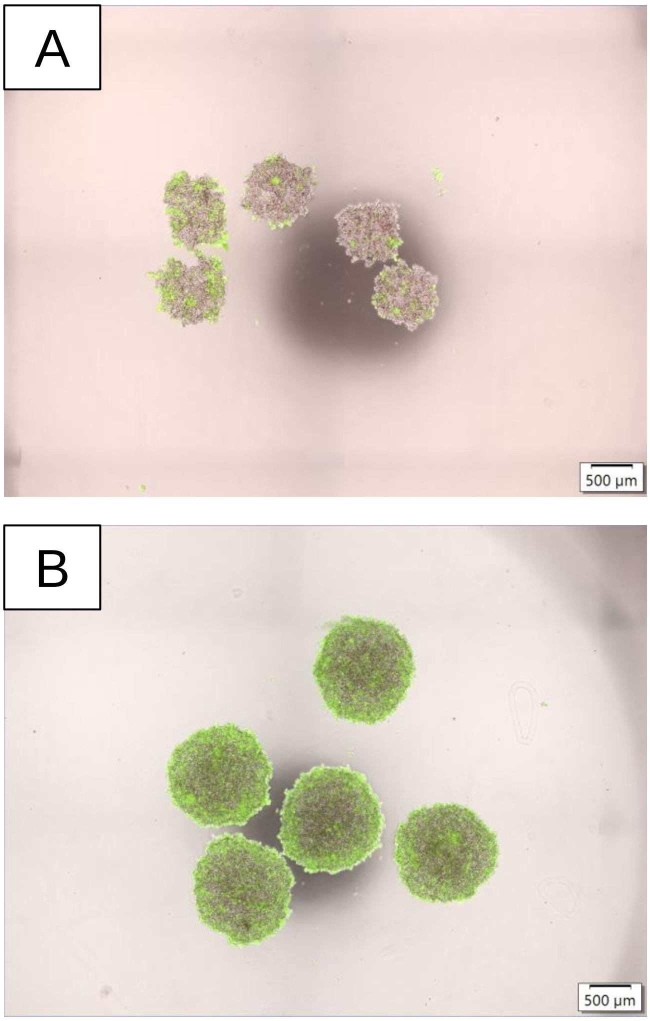

Figure S13. Evaluation of the optimal conditions for efficient attachment of hMSCs to PANC-1 spheroids.
(A) Five small PANC-1 spheroids (5×10^3 cells/spheroid) and 2.5×10^5 hMSCs were co-cultured in 125 µL of medium in low attachment plates under rotation at 36 rpm. **(B)** Five appropriate-size spheroids (2×10^4 cells/spheroid) and 2.5×10^5 hMSCs were co-cultured in 125 µL of medium in low attachment plates under rotation at 36 rpm. Scale bar represents 500 µm.

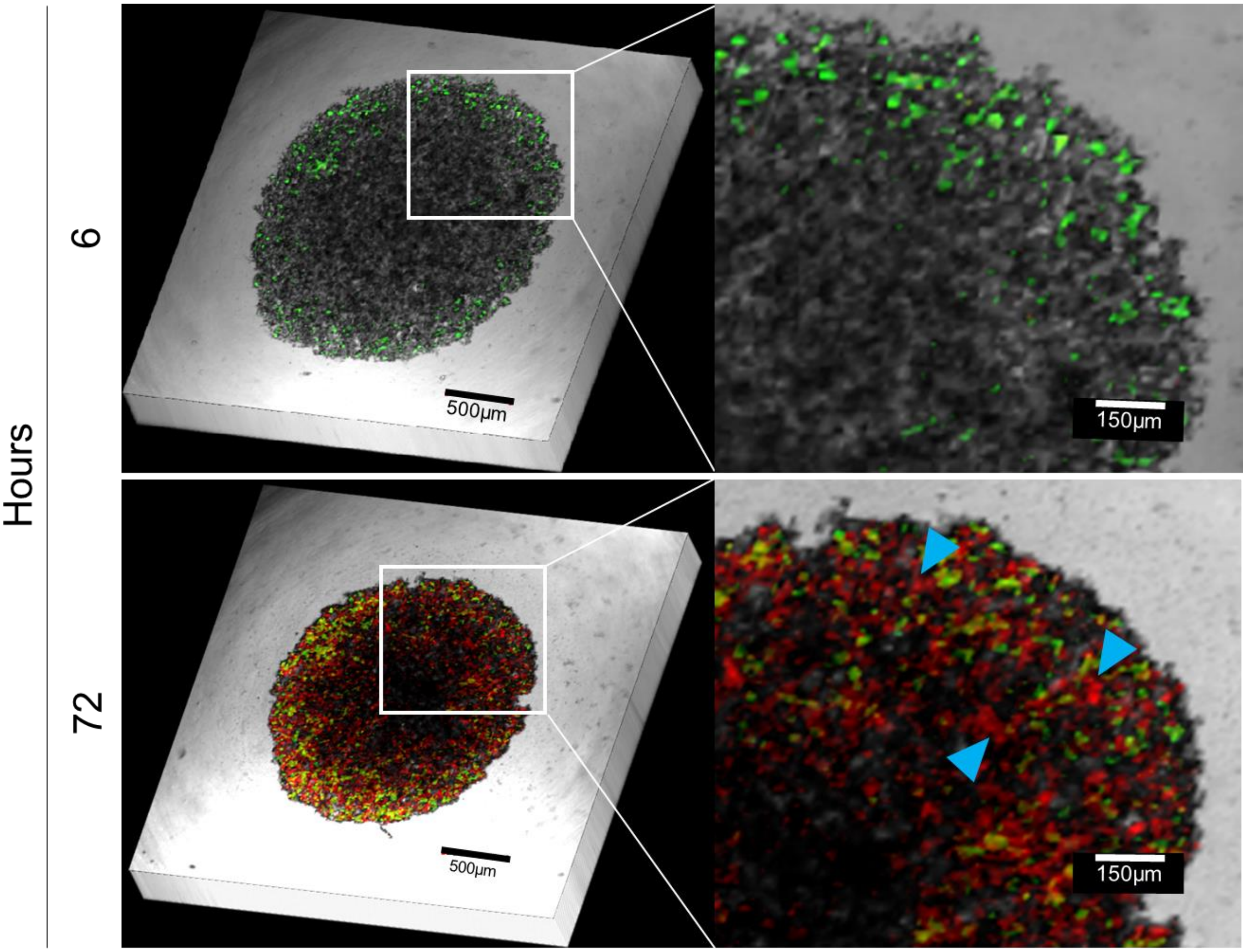

Figure S14. Spread of oHSVs in PANC-1 cells in a 3D co-culture model.
Confocal micrographs of a middle section of a PANC-1 spheroid co-cultured with oHSV-mCherry-loaded BMMSCs. The section was cut at a position of 400 µm and had a thickness of 750 µm. Scale bar represents 500 µm. Magnified images are also shown. Scale bar represents 150 µm. A PANC-1 spheroid and oHSV-BMMSCs were co-cultured under horizontal rotation and seeded in a low attachment plate. PANC-1 cells: not labeled, BMMSCs: green, mCherry: red. Blue arrowheads indicate PANC-1 cells infected with oHSV but not co-cultured with BMMSCs.

Table S1. Primers used in this study.

1	ACTCACTATAGGGCGAATTGATTTAAATGCGTGTGTTTCTGATCGACGACGCG
2	AGGCCTACTAGTGCTCAGCGTTTAAACTTATTTATTAACATCAAACACGCGC
3	CCTGCAGGCTTAAGGTTTAAACGTCTTTAATGGACCGCCCGCAGGGG
4	ACTAAAGGGAACAAAAGCTGATTTAAATTTTTCCCGACGACGACCTCGACGTT
5	GTTTAAACGCTGAGCACTAGTAGGCCTTAGTTATTAATAGTAATCAATTACG
6	GTTTAAACCTTAAGCCTGCAGGATACATTGATGAGTTTGGACAAACC
7	TAAGTTTAAACGCTGAGCACTAGTAGGCCTTAGTTAATTAAGCTGCAAGGCGATTAAGTTGGGTAA
8	GACGTTTAAACCTTAAGCCTGCAGGATATTAATTAACATGATTACGCCAAGCTCGAAATTA
9	CATGTGACTCCACGGAGTACCGGGCGCCGT
10	CTCGCCCTTGCTCACCATGGTGGCGGCTAGCGGATCCGGTGCACCTGCAGGTTCACGACACC
11	CTGCAGGTGCACCGGATCCGCTAGCCGCCACCATGGTGAGCAAGGGCGAG
12	CTATAGAATACTCAAGCTTGCATGCCAGTGAAAAAAATGCTTTATTTGTG
13	AATTCAAAATTTTATCGATACTAGTCCGTCACCACCCCCCCCAACCCGCC
14	TCGGCGCCCTTGCTCACCATGGTGGCGACTACGTAGAATTCTGGCGACCGGTAGCTCGACTCTAGA
15	AGTCGAGCTACCGGTCGCCAGAATTCTACGTAGTCGCCACCATGGTGAGCAAGGGCGCCGAGCTGT
16	TTGTAATCCAGAGGTTGATTTCGCGAGCTAGCTCACTTGTACAGCTCATCCATGCCG
17	TGGATGAGCTGTACAAGTGAGCTAGCTCGCGAAATCAACCTCTGGATTACAAAATTT
18	CTTTCCACACCGCGGCCGCGGATCCAGGCGGGGAGGCGGCCCAAAGGGAG
19	GAGCCGGTTGACTATTACGC
20	ACTCCAGGTTCTCAGGTTGG
